# CDK9 is a dependency in GATA-3 driven and MCL-1 independent T-cell Lymphomas

**DOI:** 10.1038/s41408-025-01427-1

**Published:** 2025-11-27

**Authors:** Chenguang Wang, Suhaib Abdelrahman, Xiangrong Geng, Alyssa Burgess, Ying S. Hu, Mohd Ahmar Rauf, Nermin Kady, Yao Fu, Tara A. Reilly, Ira P. Maine, Phillip Boonstra, Kirill Sabitov, Carlos Murga-Zamalloa, Ryan A. Wilcox

**Affiliations:** 1 Department of Internal Medicine, Division of Hematology and Oncology, University of Michigan, Ann Arbor, MI USA; 2 Department of Chemistry, College of Liberal Arts and Sciences, University of Illinois Chicago, Chicago, IL USA; 3 Clinical Pathology Department, Faculty of Medicine, Mansoura University, Mansoura, Egypt; 4 Department of Biostatistics, School of Public Health, University of Michigan, Ann Arbor, MI USA; 5 Department of Pathology, University of Illinois Chicago, Chicago, IL USA

**Keywords:** T-cell lymphoma, Oncogenes

## Abstract

The transcription factor GATA-binding protein 3 (GATA-3) regulates oncogenic transcriptional programs across diverse T-cell lymphomas, including subsets of both peripheral and primary cutaneous T-cell lymphomas. These GATA-3 dependent transcriptional programs, in collaboration with the genetic landscape, promote cell growth and survival, and confer resistance to conventional chemotherapeutic agents. We observed that transcriptional cyclin dependent kinase 9 (CDK9) activation regulates diverse oncogenic transcriptional programs in these aggressive T-cell lymphomas and is thus a novel therapeutic vulnerability. Using complementary and orthogonal approaches, we identified multiple independent mechanisms by which CDK9 promotes T-cell lymphomagenesis, including a mechanism by which GATA-3 promotes CDK9 activation at GATA-3 dependent loci. We also identify novel mechanisms by which GATA-3 and CDK9 regulate rRNA transcription and processing, respectively, collaboratively promoting ribosome biogenesis. Therefore, CDK9 is a therapeutic vulnerability across genetically and transcriptionally diverse T-cell lymphomas, including those for which GATA-3 is oncogenic.

## Introduction

Peripheral T-cell lymphomas (PTCL) are derived from mature, post-thymic T cells, and while they account for ≈10% of non-Hodgkin lymphomas (NHL), they increasingly account for a disproportionate number of lymphoma-related deaths, as the significant therapeutic gains achieved for the more common B-cell NHL have not yet been realized for many PTCL subtypes, as currently available therapies are rarely curative and mechanisms of treatment resistance are poorly understood [1]. In fact, the most prevalent PTCL subtype worldwide is heterogeneous and remains “not otherwise specified” (PTCL, NOS), a subset of which, despite treatment with standard frontline anthracycline-based chemotherapy, anticipate an overall survival that is not significantly different from those undergoing hospice care alone [2]. Oncogenic transcriptional programs, the genetic landscape, and the tumor microenvironment (TME) collaboratively promote PTCL pathogenesis and treatment resistance [1, 3, 4]. For example, interactions between antigen-presenting cells within the TME and malignant T cells culminate in activation of antigen- and costimulatory-receptor dependent transcriptional programs [2, 5, 6]. Gain-of-function alterations in cell surface receptors or their downstream signaling intermediates stabilize these receptor-ligand interactions or further amplify ligand-dependent signaling, respectively [7–10]. Copy number alterations affecting oncogenes (including *MYC*) and tumor suppressors (including *TP53* and *PTEN*) are recurrently observed and further promote the biomass production required for cell proliferation [11–13]. While the potential role of anti-apoptotic proteins in treatment resistance is incompletely understood, MCL-1 is highly expressed in the majority of PTCL [14], a subset of which are MCL-1 dependent [15]. Primary cutaneous T-cell lymphomas (CTCL), particularly those with large cell transformation (LCT), share transcriptional and genetic characteristics, and potential therapeutic vulnerabilities, with treatment refractory PTCL, NOS [2, 16, 17]. Collectively, these findings highlight the need for improved therapeutic strategies and further explain why clinical trial participation remains a preferred treatment for patients afflicted with these T-cell lymphomas [1, 4]. While studies investigating targeted agents have been completed, or are ongoing, few complete or durable responses are achieved. The transcriptional, genetic, and microenvironmental complexity of these lymphomas, while therapeutically challenging, might also suggest that “multitargeted” strategies converging on multiple and non-redundant dependencies may overcome the challenges historically associated with more “targeted” approaches.

Cyclin-dependent kinases (CDK’s) are commonly dysregulated in human cancers and have emerged as significant therapeutic vulnerabilities given their important roles in both cell cycle and transcriptional regulation [18–20]. For example, the efficiency of transcriptional elongation by RNA polymerase II (RNAP2) is a CDK-dependent regulatory checkpoint. Negative transcriptional elongation factors (N-TEF, including NELF and DSIF protein complexes), by pausing transcription, prevent gene expression in the absence of appropriate stimuli [21–25]. Productive transcriptional elongation at many genes requires the recruitment of the positive transcriptional elongation factor b (P-TEFb), which includes CDK9 in complex with cyclin T1 [or cyclin T2 or cyclin K [22, 26–30]]. Upon recruitment, active CDK9 phosphorylates three targets that are critically important for release of transcriptional pausing and efficient transcriptional elongation, including serine phosphorylation within the c-terminal domain (CTD) of RNAP2, leading to efficient transcriptional elongation and pre-mRNA processing [31–33]; the NelfE subunit of the NELF complex, liberating it from RNAP2 [34]; and the Spt5 subunit of the DSIF complex, converting this complex into a positive transcriptional elongation factor [35, 36]. These CDK9-dependent phosphorylation events prevent transcriptional pausing and thus promote efficient transcriptional elongation, which is a key determinant of transcriptome composition [37], and required for cell proliferation [38]. As selective CDK9 inhibitors are currently being investigated in multiple cancers [39, 40], and prior screening efforts have suggested that CDK9 is a potential therapeutic vulnerability in T-cell lymphomas [41, 42], we sought to examine its role in regulating oncogenic transcriptional programs in the T-cell lymphomas.

## Methods

### Patients and cell lines

All cell lines were mycoplasma free and independently authenticated by short tandem repeat (STR) profiling, performed by ATCC, and immunophenotyping. Primary malignant T cells for ex vivo studies were isolated from patients with Sezary syndrome, as previously described [2, 5, 6, 43].

### Genetically engineered mouse models

Mouse studies were approved by the University Committee on Care and Use of Animals (UCUCA) and performed in accordance with guidelines established by the Unit for Laboratory Animal Medicine (ULAM) at University of Michigan. Mice were housed under specific-pathogen free conditions. Treatment allocation was randomized, and all animals in given experiments were included for analysis. Floxed (SNF5, PTEN, TP53) and CD4-Cre mice were obtained from Jackson Laboratory and were crossed). All GEM models used here have been previously described [2, 44, 45]. Lymphoma burden was determined by the development of massive hepatosplenomegaly and/or bulky lymphadenopathy (>5 mm). For adoptive transfer experiment, 5 × 10^6^ bulk splenocytes obtained from lymphoma-bearing donor mice were retro-orbitally injected into 12–16 weeks old recipient C57BL/6 J mice.

### Chromatin immunoprecipitation (ChIP) and sequencing

ChIP was performed using SimpleChIP^®^ Plus Enzymatic Chromatin IP Kit (Cell Signaling #9004) with minor modifications. Briefly, 10 µg chromatin was incubated with primary antibody overnight. Chromatin was then incubated with 30 µL Dynabeads Protein G (Invitrogen #10004D) for 4 h and washed with low-salt washing buffer three times and high-salt washing buffer once. Eluted samples were purified by QIAquick^TM^ PCR Purification Kit (Qiagen #28104) and were used for ChIP-seq library construction, according to NEBNext ChIP-Seq Library Prep Master Mix Set for Illumina (NEB #E6240) manual instructions. Library was sequenced on the Illumina NovaSeq X plus platform.

### Statistical analysis

GraphPad Prism 10.0 and SPSS 13.0 software were used for data analysis. All Animal experiments were analyzed by independent *t*-tests (two-tailed) for tumor volume, tumor weight, tumor cells. Survival analysis was done by Wilcoxon test. Correlation was analyzed by two-tailed person correlation coefficients. All other analysis were done using Welch’s unpaired *t*-test. Additional details about methods and analyses are provided in the Supplemental Methods.

## Results

### CDK9 is a therapeutic vulnerability in highly proliferative T-cell lymphomas

**Fig. 1:**
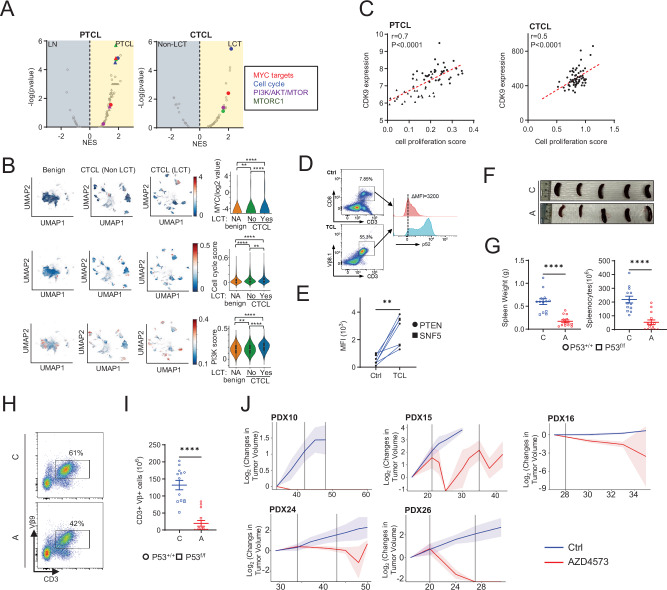
CDK9 is a therapeutic vulnerability in T-cell lymphomas. **A** Gene set enrichment analysis (GSEA) was performed using PTCL (accession number: GSE36172, shown in circles and GSE160119, shown in triangles) and CTCL RNA-seq datasets. **B** Uniform manifold approximation and projection (UMAP) showing MYC expression, G2M checkpoints and PI3K pathway (from MSigDB Hallmark database) enrichment scores in benign skin and CTCL, stratified by LCT status, in compiled scRNA-seq data sets. Scores were also summarized as violin plots on the right. **C** Cell proliferation enrichment score was generated and correlated with normalized CDK9 expression in public PTCL and in-house MF RNA-seq datasets. Two PTCL databases (accession number: GSE36172 and GSE160119) were shown in circles and triangles, respectively. **D, E** Representative (**D**) and summarized (**E**) phospho-RNAP2 (pS2) expression by flow cytometry in non-clonal CD3^+^CD8^+^ T-cells from C57BL/6 J (Ctrl) mice and clonal CD3^+^Vβ8.1^+^ T-cell lymphoma cells from p53^fl/fl^ CD4-Cre^+^ mice (TCL, *n* = 4, circles) and PTEN^fl/fl^ CD4-Cre^+^ mice (TCL, *n* = 3, squares), respectively. **F**, **G** Splenocytes from lymphoma-bearing p53^fl/fl^PTEN^fl/fl^CD4-Cre^+^ or p53^+/+^PTEN^fl/fl^CD4-Cre^+^ were adoptively transferred into syngeneic B6 recipients (*n* = 4–5 recipients/experimental group). Upon lymphoma engraftment, mice were treated with either vehicle control (C) or AZD4573 (A) by intraperitoneal injection (15 mg/kg, twice daily for 2 consecutive days weekly with two hours interval for 1–3 weeks). Representative examples are shown in (**F**) and spleen weights summarized in (**G**) stratified by P53 status. Data was combined from 3 biological replicates. **H, I** Clonal T cell (CD3^+^ Vβ9^+^) abundance and cell proliferation (by Ki67 expression) were quantified in spleens of vehicle control (C) or AZD4573 (A) treated syngeneic recipient mice. Representative examples are shown in (**H**) and the number of cells summarized in (**I**). Data was combined from 3 biological replicates. **J** Independent PTCL, NOS PDX (*n* = 5) were established subcutaneously in NSG recipients (*n* = 4–5/treatment group). Upon engraftment, PDX were randomized to treatment with vehicle control (Ctrl, blue) or AZD4573 (red), as before. Changes in tumor volume were assessed at each observed timepoint with the baseline (first measurement), plotted as log₂ fold-change trajectories with shaded ribbons indicating ±1 standard deviation across samples. Gray vertical lines indicate day of treatment. Data are represented as mean ± SEM and were analyzed using Welch’s unpaired *t*-test. Correlation was analyzed by two-tailed pearson correlation coefficients. **p < 0.01; ***P < 0.001; ****P < 0.0001.

Oncogenic transcriptional programs were examined in PTCL and CTCL with LCT, using reactive lymph nodes and CTCL without LCT (non-LCT) as controls, respectively. Transcriptional programs associated with c-myc, PI3K/AKT/mTOR, and cell proliferation were observed in PTCL and in CTCL with LCT in independent bulk RNA-seq (Fig. 1A) and scRNA-seq (Fig. 1B) datasets. These transcriptional programs, prevalent in T-cell lymphomas [16, 46, 47], were directly associated with both CDK9 expression (Supplemental Fig. 1A) and cell proliferation (Supplemental Fig. 1B). Transcriptional data were utilized to generate a composite cell proliferation score (Fig. 1C), and this too was highly associated with CDK9 expression. Strong CDK9 expression was detected by immunohistochemistry in 90% of human T-cell lymphomas examined (Supplemental Fig. 1C). Upon activation, CDK9 phosphorylates the c-terminal tail of RNAP2. Therefore, CDK9-dependent RNAP2 phosphorylation was examined in patient-derived cell lines, and constitutive CDK9 activation uniformly observed (Supplemental Fig. 1d). As components of the SWI/SNF complex, TP53, and PTEN are recurrently mutated and/or deleted in diverse T-cell lymphomas, CDK9-dependent RNAP2 phosphorylation was similarly examined in representative GEM models that spontaneously develop T-cell lymphomas [2, 45]. In contrast to normal (non-clonal) T cells, a significant increase in RNAP2 phosphorylation was observed in lymphoma cells (Fig. 1D, E). In order to directly examine the extent to which cell proliferation is dependent upon CDK9 activity, lymphoma-bearing, including p53-deficient, mice were treated with the selective CDK9 inhibitor AZD4573 [40]. A significant reduction in spleen weight and cellularity was observed (Fig. 1F, G), irrespective of p53 status, and this was associated with a significant reduction in both total and proliferative TCR-Vβ+ clonal T cells (Fig. 1H, I). Similar results were obtained in an independent murine model (Supplemental Fig. 1E–H). In conjunction with prior screening efforts [41, 42], this data suggests that CDK9 is a viable therapeutic vulnerability in genetically high-risk T-cell lymphomas. In order to examine this further, five independent PTCL, NOS patient-derived xenografts (PDX) were generated and treated with AZD4573 or vehicle control. A significant decrease in tumor volume (Fig. 1J and Supplemental Fig. 1I–J) was observed in animals treated with AZD4573 in all five PDX models. This observation was further confirmed via a generalized linear mixed-effects model (GLMM) with log-tumor volume as the outcome. Without treatment, tumors grew at an estimated average rate of 0.116 log-mm^3^ per day (95% CI: [0.043, 0.189]; *p* = 0.002), corresponding to 12.2% day-over-day growth. Upon receiving one AZD4573 treatment, average growth decreased relative to untreated mice by an estimated 0.281 log-mm^3^ per day (95% CI: [−0.339, −0.212]; *p* < 0.001), translating to an overall estimated decrease in tumor volume of 0.159 log-mm^3^ per day after initial treatment (Supplemental Fig. 1K).

### MCL-1 is sufficient, but not necessary, for CDK9 dependence in T-cell lymphomas

**Fig. 2:**
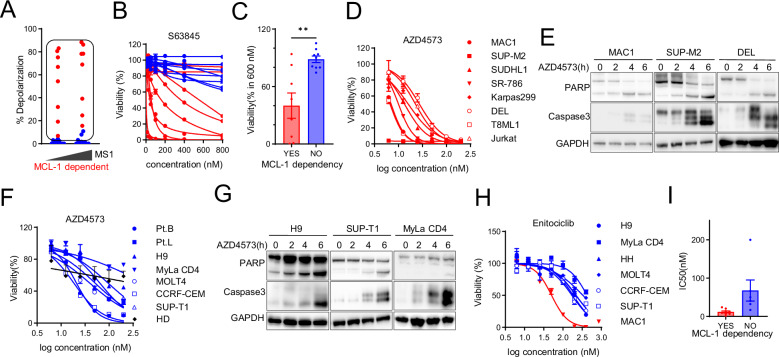
CDK9 is a vulnerability in MCL-1 independent T-cell lymphomas. **A** BH3 profiling was performed using a MCL-1 selective only BH3 peptide (MS1; 5, 10 μM) in T-cell lymphoma cell lines. Cell lines with loss of cytochrome c, measured by percentage (%) of depolarization, in the presence of MS1, indicating MCL-1 dependence, are highlighted in red. MCL-1 dependent cell lines were identified if their percentage of depolarization exceed 10% or if they exhibit higher depolarization in higher concentration of MS1 peptide. **B**, **C** Cell viability was determined in a panel of cell lines treated with a MCL-1 selective inhibitor (S63845). MCL-1 dependent and independent cells are indicated in red and blue, respectively. Cell viability at 600 nM is summarized in (**C**) stratified by MCL-1 dependence. **D** Cell viability was determined in MCL-1 dependent cell lines treated with AZD4573 (2-fold serial dilution from 200 nM for 24 h). **E** Apoptosis was measured by cleaved PARP and Caspase-3 IB in representative MCL-1 dependent (MAC1, SUP-M2 and DEL) cells. **F** Cell viability was determined in MCL-1 independent cell lines, SS specimens and normal T cells (HD) from healthy donors (*n* = 3) treated with AZD4573 (2-fold serial dilution from 200 nM for 24 h). **G** Apoptosis was measured by cleaved PARP and Caspase-3 IB in representative MCL-1 independent (H9, MyLa CD4, SUP-T1) cells. **H** Cell viability was determined in MCL-1 dependent (in red) and independent (in blue) cell lines, treated with selective CDK9 inhibitor Enitociclib (2-fold serial dilution from 400 nM for 24 h). **I** IC50 of AZD4573 is shown in each cell line stratified by MCL-1 dependency. Data are represented as mean ± SEM and were analyzed using Welch’s unpaired *t*-test. **p < 0.01.

As MCL-1 transcripts are short-lived, the maintenance of MCL-1 expression is dependent upon efficient transcriptional elongation and CDK9 activation, and thus CDK9-dependent MCL-1 transcription has been identified as a significant mechanism of action for CDK9 antagonism in MCL-1 dependent hematologic malignancies [40]. The extent to which MCL-1 dependence confers sensitivity to CDK9 antagonism was examined next, as a subset of T-cell lymphomas are MCL-1 dependent [15]. Not surprisingly, we observed a significant reduction in MCL-1 expression upon CDK9 antagonism (Supplemental Fig. 2A). In order to determine the extent to which MCL-1 loss is a significant mechanism of action for CDK9 antagonism in the T-cell lymphomas, BH3 profiling was performed and cell lines stratified by MCL-1 dependence (Fig. 2A). These results were pharmacologically validated (Fig. 2B, C) using a selective MCL-1 antagonist [S63845 [48]]. As anticipated, MCL-1 dependent T-cell lymphomas were highly sensitive to CDK9 antagonism with AZD4573 (Fig. 2D), culminating in PARP and caspase 3 cleavage within 6 h of treatment (Fig. 2E). However, we also observed that MCL-1 independent T-cell lymphoma cells were also sensitive to both AZD4573 (Fig. 2F, G) and an independent CDK9 antagonist [enitociclib [49], (Fig. 2H)]. While the IC_50_ for AZD4573 was lower in MCL-1 dependent cells (Fig. 2I, Supplemental Table S1), it should also be noted that the IC_50_ for MCL-1 independent cells was in the low nanomolar range (≈50 nM), suggesting that while MCL-1 dependence may be sufficient for sensitivity to CDK9 antagonism, it is not necessary. In contrast, the IC_50_ for normal (non-malignant) T cells obtained from normal donors was not reached (Fig. 2F).

### The GATA-3 dependent transcriptome is CDK9 dependent

**Fig. 3:**
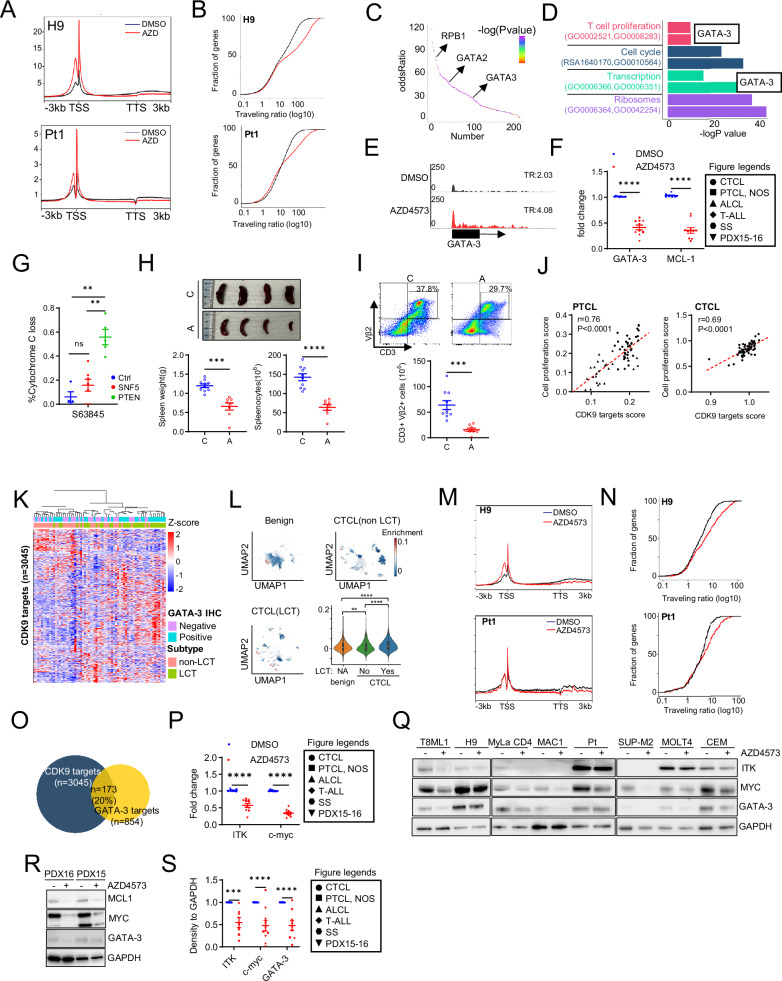
GATA-3 and GATA-3 dependent transcriptome are CDK9 dependent. **A** Genome-wide average RNAP2 ChIP-seq profile is shown in H9 cells and a PTCL, NOS patient specimen (Pt1) treated with DMSO (black) or AZD4573 (red: 50 nM for 4 h). **B** Distribution of RNAP2-bound genes with a given traveling ratio in H9 and Pt1 cells treated with DMSO (black) and AZD4573 (red: 50 nM for 4 h). **C** Genes that have increased traveling ratio from ChIP-seq were selected to analyze their overlap with public transcription factor (TF) -regulated genomic regions. Overlapped TF were ordered by odds ratio and significance. **D** Integrated analysis ChIP-seq and RNA-seq was performed in cells (H9) treated with AZD4573 or vehicle control and CDK9 dependent genes was identified, and representative enriched gene sets were showed by category. **E** Representative RNAP2 ChIP-seq signal on GATA-3 locus in H9 cells treated with AZD4573. Traveling ratio (TR) is shown at right. **F** MCL-1 and GATA-3 were identified as CDK9 targets and were validated by qRT-PCR in DMSO (blue) and AZD4573 (red) treated CTCL (H9, MyLa CD4, MAC1), ALCL (SUP-M2), T-ALL (SUP-T1), PTCL, NOS (T8ML1), SS specimens and PDX cells. **G** MCL-1 dependence was determined by BH3 profiling using a selective MCL-1 inhibitor (S63845) in normal (non-clonal) T-cells obtained from B6 mice and in clonal lymphoma cells obtained from SNF5^fl/fl^CD4-Cre^+^ and PTEN ^fl/fl^CD4-Cre^+^ mice, as indicated. **H** Splenocytes from lymphoma-bearing p53^fl/fl^SNF5^fl/fl^CD4-Cre^+^ mice (2 biologic replicates) were adoptively transferred into syngeneic B6 recipients (*n* = 4 recipients/experimental group). Upon lymphoma engraftment, mice were treated with either vehicle control (**C**) or AZD4573 (**A**). At the time of study termination, spleens were explanted and weighed. Representative examples are shown on the top, and the data summarized on the bottom. **I** Clonal T cells (CD3^+^ Vβ2^+^) in spleens were quantified in vehicle control (**C**) or AZD4573 (**A**) treated mice. Representative examples are shown (top) and the data summarized (bottom). **J** Correlation of enrichment score for CDK9 target genes and cell proliferation enrichment score is shown in public PTCL and in-house MF gene expression profiling datasets. Two PTCL databases (accession number: GSE36172 and GSE160119) were shown in circles and triangles, respectively. **K** Heat map showing CDK9 target genes (*n* = 3045) in unsupervised clustering of an in-house MF cohort stratified by LCT status. **L** Uniform manifold approximation and projection (UMAP) showing CDK9 target genes enrichment score in benign skin and CTCL, stratified by LCT status, in compiled scRNA-seq data sets. Scores were also summarized as violin plots. **M** Average RNAP2 ChIP-seq profile of GATA-3 target genes is shown in H9 cells and PTCL, NOS patient specimen (Pt1) treated with DMSO (black) and AZD4573 (red: 50 nM for 4 h). **N** Distribution of GATA-3 target genes with a given traveling ratio in H9 cells and PTCL, NOS patient specimens (Pt) treated with DMSO (black) and AZD4573 (red: 50 nM for 4 h). **O** Venn diagram showing overlap of CDK9 and GATA-3 target genes. **P** Representative GATA-3 target genes (ITK, c-myc) were validated by qRT-PCR in DMSO (blue) and AZD4573 (red) treated CTCL (H9, MyLa CD4, MAC1), ALCL (SUP-M2), T-ALL (SUP-T1), PTCL, NOS (T8ML1), SS specimens and PDX cells. **Q**–**S** Expression of GATA-3, ITK, and c-myc was determined by IB in AZD4573 treated cell lines (Q, 50 nM for 6 h), a SS patient (Pt) specimen (Q, 50 nM for 6 h) and freshly excised PDX cells (R, 4 h after last dose of treatment). MCL-1 was utilized as a pharmacodynamic biomarker in PDX mice treated with AZD4573. Density was normalized to GAPDH and summarized in (**S**). Data are represented as mean ± SEM and were analyzed using Welch’s unpaired *t*-test. Correlation was analyzed by two-tailed pearson correlation coefficients. **p < 0.01; ***P < 0.001; ****P < 0.0001; ns not significant.

In order to explore MCL-1 independent mechanisms, a representative cell line (H9) and primary patient specimen were treated with AZD4573 or vehicle control and RNAP2 ChIP-seq performed. RNAP2 accumulation adjacent to the transcriptional start site was observed (Fig. 3A, Supplemental Fig. 3A), and an increase in RNAP2 traveling ratios (TRs), consistent with impaired transcriptional elongation, observed across ≈50% of the transcriptome (Fig. 3B). Compatible with these findings, significant alterations in the transcriptome were observed by bulk RNA-seq upon AZD4573 treatment (Supplemental Fig. 3B). As CDK9 may collaborate with other transcriptional regulators, transcription factor motif analysis was performed among differentially expressed transcripts, and enrichment in GATA-3 binding motifs observed (Fig. 3C). This was notable, as GATA-3 expression identifies PTCL and CTCL that are clinically, genetically, and transcriptionally distinct, owing to their dependence upon a GATA-3-driven transcriptional program and a genetic landscape associated with increased biomass production suitable to meet the demands of these highly proliferative T-cell lymphomas [2, 5, 11, 50, 51]. Furthermore, and consistent with our data implicating CDK9 in proliferation and biomass production (Fig. 1A–C, H, I), significant reductions in gene sets associated with these biological processes were observed upon CDK9 antagonism (Fig. 3D, Supplemental Fig. 3C), many of which included GATA-3 (Fig. 3D). Therefore, RNAP2 pausing at the GATA-3 locus upon CDK9 antagonism was examined (Fig. 3E), and significant transcriptional pausing observed. The abundance of GATA-3 transcripts was examined across diverse PTCL/CTCL cell lines and primary specimens upon CDK9 antagonism with AZD4573. As MCL-1 is highly CDK9 dependent [40], MCL-1 was examined as a positive control. Importantly, a significant reduction in GATA-3 transcription, comparable to the reduction observed for MCL-1, was observed upon CDK9 antagonism (Fig. 3F). Therefore, we sought to examine the extent to which CDK9 antagonism is a therapeutic vulnerability in vivo using a GATA-3 dependent, but MCL-1 independent, T-cell lymphoma model. We pharmacologically examined the extent to which the T-cell lymphomas that emerge upon conditional loss of *SMARCB1* (SNF5) are MCL-1 dependent (Fig. 3G). We had previously shown that GATA-3 is oncogenic in this GEM model and demonstrate here that these T-cell lymphomas are MCL-1 independent (Fig. 3G). Therefore, lymphoma-bearing mice were treated with AZD4573 or vehicle control, and a significant reduction in disease burden was observed (Fig. 3H, I). In a model cell line, CDK9 antagonism led to transcriptional pausing at 12,998 gene loci and was associated with a significant decrease in gene expression (by RNA-seq) at 3045 gene loci (Supplemental Fig. 3D). Similar to CDK9 itself, these CDK9 dependent genes were also associated with cell proliferation (Fig. 3J). Therefore, we examined the extent to which these CDK9 dependent genes are enriched in CTCL with LCT in independent bulk and scRNA-seq datasets. CDK9 dependent genes were enriched in CTCL with LCT, most of which highly express GATA-3 (Fig. 3K–L; Supplemental Fig. 3E), and a similar enrichment in CDK9 dependent genes was observed in PTCL (Supplemental Fig. 3E). Therefore, we next examined the extent to which GATA-3 target genes, which we had previously identified [2], are CDK9 dependent. Consistent with the genome wide analyses, significant transcriptional pausing was observed upon CDK9 antagonism at many GATA-3 dependent loci (Fig. 3M, N, Supplemental Fig. 3F). Overall, transcriptional pausing was observed at 20% of GATA-3 target genes (Fig. 3O). In order to validate these findings, we selected two clinically relevant GATA-3 target genes [ITK [2, 5] and c-Myc [2, 11]] for validation. In both cell lines and primary specimens, a significant reduction in ITK and c-Myc transcripts (Fig. 3P) and protein (Fig. 3Q–S) were observed upon CDK9 antagonism. Efficient transcription of GATA-3 itself, and a substantial subset of its target genes, are reliant on CDK9.

### GATA-3 binds and recruits CDK9

**Fig. 4:**
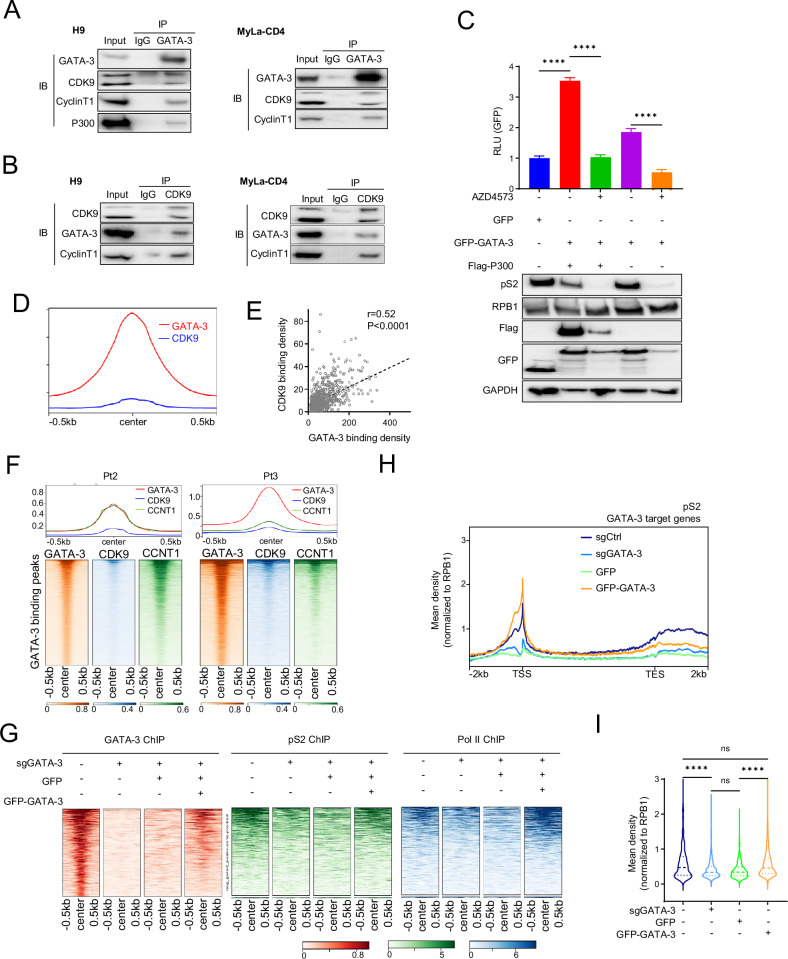
GATA-3 binds and recruits CDK9. **A, B** Endogenous GATA-3 and CDK9 are co-immunoprecipitated, as indicated, in H9 and MyLa CD4 cell lines. GATA-3 immunoprecipitation (IP) and CDK9 IP are shown in (**A**) and (**B**), respectively. **C** Relative luciferase activity in HEK293T cells expressing a GATA-3-dependent reporter and GFP-tagged GATA-3 and/or Flag-tagged P300 treated with AZD4573 or vehicle control, as indicated. Protein expression is examined by IB, and the corresponding blots are shown. **D** Average ChIP-seq peaks of GATA-3 and CDK9 around GATA-3 binding sites. **E** Correlation of GATA-3 and CDK9 binding density at all GATA-3 binding sites. **F** Average TSS ChIP-seq profiles for GATA-3, CDK9 and CCNT1 and metagene occupancy heatmaps for GATA-3 binding sites in two PTCL, NOS patient specimens. **G** Metagene occupancy heatmaps for GATA-3 binding sites were shown in GATA-3, RNA pol II and phosphor-rRNA pol II (pS2) ChIP-seq profiles of control, GATA-3 KO (sgGATA-3), GATA-3 KO transfected with GFP (GFP) and GATA-3 KO transfected with wild type GATA-3 (GFP-GATA-3) H9 cells. **H** Average profile of pS2 in GATA-3 target genes was shown in control (sgCtrl), sgGATA-3, GFP and GFP-GATA-3 H9 cells. Density of pS2 were normalized to total RPB1 density on matched conditions. **I** pS2 ChIP-seq signal (normalized to RNAP2 ChIP-seq) at GATA-3 target genes is summazied in sgCtrl, sgGATA-3, GFP, GFP-GATA3 H9 cells. Data are represented as mean ± SEM and were analyzed using Welch’s unpaired *t*-test. Correlation was analyzed by Pearson correlation coefficients. ****P < 0.0001; ns not significant.

Transcription factors may recruit CDK9 to their target genes [52–56]. GATA-1, for example, binds cyclin T1 and promotes CDK9 activation [57]. Therefore, we entertained the hypothesis that GATA-3 may similarly bind CDK9 (and/or cyclin T) and promote CDK9 recruitment to its target genes. In immunoprecipitation studies, both CDK9 and cyclin T1 were pulled down with GATA-3 (Fig. 4A) while GATA-3 was pulled down with CDK9 (Fig. 4B). We had previously shown that p300 dependent GATA-3 acetylation is required for optimal DNA binding and target gene expression [2]. In a GATA-3 dependent luciferase reporter assay, CDK9 antagonism significantly impaired reporter gene expression independently of p300 (Fig. 4C), and CDK9 antagonism did not impair protein-protein interactions between GATA-3 and p300 (Supplemental Fig. 3G). While CDK9 is not thought to bind DNA directly, presumed indirect DNA interactions were observed by ChIP-seq in both cell lines (H9) and patients (Pt2, Pt3), as CDK9 binding peaks aligned with GATA-3 binding peaks (Fig. 4D–F), including previously identified GATA-3 target genes (Supplemental Fig. 3H–I). In order to examine the extent to which GATA-3 may recruit CDK9 to its target genes, we generated GATA-3 knock out (KO) cells by gene editing and performed GATA-3, RNAP2 (pS2) and RNAP2 ChIP-seq (Fig. 4G, Supplemental Fig. 3J). A significant decrease in RNAP2 (pS2) and RNAP2 binding was observed at GATA-3 target genes upon GATA-3 KO, but was restored with the “add back” of a GFP-tagged GATA-3 (Fig. 4G, I, Supplemental Fig. 3K). The presence of GATA-3 was associated with CDK9 activation, as RNAP2 (pS2) binding peaks, after normalizing for total RNAP2 binding, remained GATA-3 dependent (Fig. 4H, I). These data indicate that GATA-3 is required for efficient CDK9 recruitment and activation at GATA-3 target genes.

### CDK9 regulates rRNA processing and ribosome biogenesis in GATA-3 dependent T-cell lymphomas

**Fig. 5:**
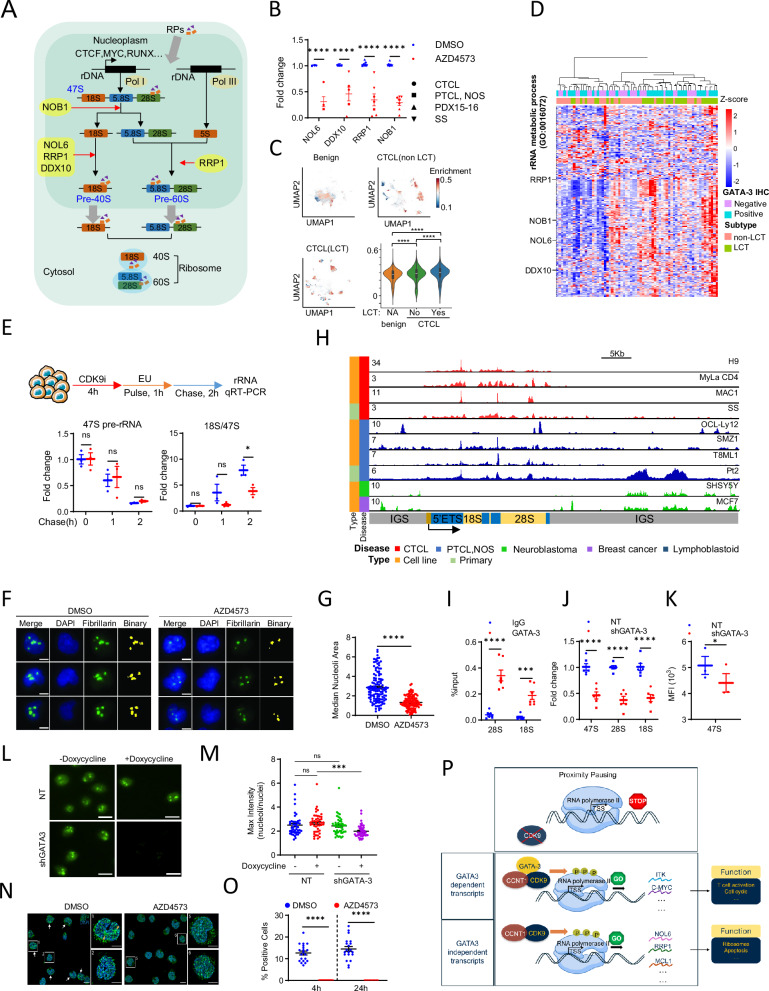
CDK9 regulates ribosome biogenesis. **A** Representative diagram of ribosome biogenesis in eukaryotes. Representative genes regulated by CDK9 involved in rRNA processing are highlighted in yellow. **B** Representative CDK9 dependent rRNA processing genes (NOL6, NOB1, DDX10, RRP1) were quantified by qRT-PCR in DMSO (blue) and AZD4573 (red) treated CTCL (H9, MAC1), PTCL, NOS (T8ML1), SS specimens and PDX models. **C** Uniform manifold approximation and projection (UMAP) showing enrichment score of genes in rRNA metabolic process in benign skin and CTCL, stratified by LCT status, in compiled scRNA-seq data sets. Scores were also summarized as violin plots. **D** Heatmap showing genes in rRNA metabolic process (*n* = 265) in unsupervised clustering of an MF cohort stratified by LCT status. **E** Cell-population-based RNA pulse-chase assays were used to assess pre-rRNA synthesis (47S pre-rRNA) and processing (ratio of 18S expression compared to 47S expression) following 4 h of CDK9 inhibition. **F**, **G** Representative images of DAPI and fibrillarin staining shown in H9 cells treated with 50 nM AZD4573 for 2 h (**F**). Binary mask was shown as median nucleoli area (fibrillarin positive regions) and was summarized in (**G**). Scale bar is 5 µm. **H** Representative GATA-3 ChIP-seq peaks on rDNA genomic loci in CTCL, PTCL, NOS cell lines and primary samples. GATA-3 ChIP-seq from neuroblastoma and breast cancer cells were used as negative control. **I** GATA-3 binding on rDNA genomic loci were validated by ChIP-qPCR in H9 (circle) and MAC1 (rectangle) cell lines. **J** 47S pre-rRNA and mature rRNA expression (18S and 28S) were measured by qRT-PCR in non-targeting (NT) and GATA-3 knock down (shGATA-3) cells (left). **K** 47S pre-rRNA was measured by RNA FISH in non-targeting (NT, blue) and GATA-3 knock down (shGATA-3, red) cells. **L**, **M** Representative images of RPL7 staining shown in negative control (NT) and GATA-3 knock-down (shGATA-3) H9 cells (**L**). Binary mask was shown as maximum nucleoli area per nuclei and was summarized in (**M**). Scale bar is 15 µm. **N**, **O** Representative z-stacks of DeepSIM images of RPL7 and DAPI staining shown in H9 cells treated with 50 nM AZD4573 for 4 and 24 h (**N**). Representative cells expressing RPL7 are indicated with white arrows. Scale bar is 10 µm. Subpanel 1 shows a representative cell with intranucleolar RPL7 aggregates, and is indicated with orange arrows. Subpanels 2, 5 and 6 show cells without nucleolar RPL7 localization. Scale bar is 5 µm. Cells with intranucleolar RPL7 aggregation, compatible with active RiBi, were quantified in 20 regions of interest (ROIs) by widefield imaging, and the data summarized in (**O**). **P** GATA-3 dependent and independent transcriptional programs are regulated by CDK9. Efficient transcriptional elongation at the GATA-3 locus is regulated by CDK9, but GATA-3 also binds CDK9 directly, and promotes efficient transcription at many GATA-3 target genes. In addition, GATA-3 independent transcriptional programs, including those regulating rRNA processing and apoptosis, are CDK9 dependent in T-cell lymphomas. Data are represented as mean ± SEM and analyzed by Welch’s unpaired *t*-test or Wilcox’s paired *t*-test. *P < 0.05; ***P < 0.001; ****P < 0.0001; ns not significant.

CDK9 dependent transcriptional programs that are not dependent on GATA-3 may be significantly enriched in GATA-3 dependent T-cell lymphomas. For example, ribosome biogenesis (RiBi) is a therapeutic vulnerability in the T-cell lymphomas (Supplemental Fig. 4A) [45]. RiBi is a dynamic, highly regulated, and energy-consuming process involving the coordinated synthesis, processing, and assembly of 4 rRNA and 80 ribosomal proteins (RP) into the 80S ribosome (Fig. 5A). Hyperactive RiBi is a characteristic of, and a therapeutic vulnerability in, many cancers [reviewed in [58]]. More specifically, the genetic landscape [e.g., PTEN loss, c-Myc amplifications [11]] and signaling pathways [e.g., the T-cell receptor [5, 59]] characteristic of GATA-3 driven T-cell lymphomas are associated with significant translational output and hyperactive RiBi [58, 60]. The production and processing of rRNA transcripts in the nucleolus is transcriptionally regulated [61] and is sufficient to orchestrate all aspects of RiBi [62]. Therefore, it is notable that transcriptional programs related to rRNA processing and RiBi were significantly impaired upon CDK9 antagonism, including NOB1, NOL6, RRP1, and DDX10 (Fig. 5B, Supplemental Fig. 4B), which are required for efficient rRNA processing (Fig. 5A) [63]. Transcripts associated with rRNA processing or RiBi were dependencies in many T-cell lymphoma cell lines (Supplemental Fig. 4A) and were highly enriched in both CTCL (Fig. 5C, D) and PTCL (Supplemental Fig. 4C). Given the long half-life of mature rRNA transcripts, we performed a pulse-chase experiment in cells pre-treated (4 h) with AZD4573 and quantified both 47S pre-rRNA and mature (processed) 18S rRNA (Supplemental Fig. 4E). A significant difference in 47S pre-rRNA was not observed, whereas a significant decrease in the 18S/47S pre-rRNA ratio was observed (Fig. 5E), compatible with diminished rRNA processing upon CDK9 antagonism. Diverse stressors, including pharmacologic inhibition of rRNA transcription or RiBi, induce “nucleolar stress”, a hallmark of which is a redistribution of nucleolar proteins [64], including fibrillarin, which becomes less dispersed and condenses in cells undergoing nucleolar stress [64]. Therefore, fibrillarin distribution was examined after brief (2 h) exposure to AZD4573 (Fig. 5F, G, Supplemental Fig. 5F) and fibrillarin condensation, compatible with nucleolar stress, observed. As nucleolar stress is anticipated to precede diminished RiBi, we sought to examine RiBi directly. However, we were mindful that hematopoietic transcription factors may regulate rRNA transcription and RiBi [65], and a role for GATA-3 in RiBi has not been previously examined. Therefore, we examined GATA-3 binding at rDNA loci by ChIP-seq, observing multiple binding peaks across the 18S rDNA locus (Fig. 5H), in a pattern resembling that previously described for GATA-2 in alternative cellular contexts [65]. Targeted GATA-3 ChIP-qPCR (Fig. 5I) validated these findings, as did the observation that inducible GATA-3 knockdown in cell lines significantly reduced 47S pre-rRNA, 18S, and 28S rRNA transcription (Fig. 5J, K). In order to examine RiBi more directly, we performed Deep Structured Illumination Microscopy (DeepSim) to visualize ribosomal protein L7 (RPL7) aggregates. GATA-3 knockdown led to a significant reduction in RPL7 aggregates (Fig. 5L, M), without altering total RPL7 expression (Supplemental Fig. 4G). RiBi was similarly visualized upon CDK9 antagonism, and a significant reduction in RiBi observed (Fig. 5N, O). The nearly complete loss of RPL7 aggregation, required for RiBi, was comparable to that observed with the ribosomal RNA synthesis inhibitor pidnarulex (Supplemental Fig. 4H–I). Therefore, GATA-3 dependent and GATA-3 independent transcripts collaboratively promote RiBi in T-cell lymphomas by promoting rRNA transcription and processing, respectively. Collectively then, we have identified novel and complementary GATA-3 dependent and independent mechanisms by which CDK9 regulates the growth and survival of GATA-3 driven T-cell lymphomas (Fig. 5P).

## Discussion

Peripheral and advanced-stage cutaneous T-cell lymphomas increasingly account for a disproportionate number of lymphoma-related deaths. Therapeutic gains have languished behind those achieved for the more common B-cell lymphomas, as few complete and durable responses are achieved with available targeted agents [1, 50, 51, 66–68], particularly among those for which a GATA-3 dependent transcriptional program is oncogenic [2, 5]. The significant transcriptional and genetic heterogeneity observed across the T-cell lymphoma spectrum has impeded progress [1]. However, improved molecular classification of these heterogenous lymphomas, including the identification of therapeutically targetable oncogenic drivers, is a rational “divide and conquer” therapeutic strategy [4].

Among GATA-3 dependent T-cell lymphomas, the GATA-3 dependent transcriptome includes receptors, kinases, and transcription factors that play a central role in oncogenic signaling pathways, including those associated with T-cell receptor and PI3K/AKT signaling [2, 51]. In addition, GATA-3 dependent cytokines have important non-cell-autonomous roles, including promoting the expansion and polarization of lymphoma-associated macrophages [50], which are a dependency [6, 69], and associated with poor outcomes [70], in these lymphomas. In addition, these lymphomas are also genetically distinct, as PTEN loss and c-Myc gains are recurrently observed [11]. Increased RiBi, required to keep pace with translational demands, plays an essential role in malignant transformation [58], and was first recognized in a c-Myc driven B-cell lymphoma model [71]. PI3K/AKT and c-Myc activation, as observed in GATA-3 driven T-cell lymphomas, likely promote ribosome biogenesis [71–75].

The observation that GATA-3 binds rDNA loci and promotes rRNA transcription is significant, suggesting that rRNA transcription mechanistically links GATA-3 with its associated genetic landscape that is reliant on hyperactive RiBi. We have shown that expression of selected rRNA processing genes is dependent upon CDK9 activation. Consequently, CDK9 antagonism, by impairing rRNA processing, rapidly culminates in nucleolar stress, and the subsequent termination of RiBi, providing a novel MCL-1 independent mechanism by which CDK9 antagonism is a therapeutic vulnerability in GATA-3 dependent T-cell lymphomas. By also impairing GATA-3 expression, CDK9 antagonism seemingly converges on RiBi by independent mechanisms affecting both rRNA transcription and processing. CDK9 may also regulate rRNA transcription and processing by alternative mechanisms that were not examined here, but may merit future study [76, 77].

Previous strategies to directly or indirectly target GATA-3 and the transcriptional program it instigates have included NFκB antagonism, as NFκB is required for efficient GATA-3 transcription [5, 78]. This is notable, as NFκB recruits the CDK9/cyclin T1 complex to gene promoters in other cellular contexts [52], but was not examined here. Alternatively, post-translational acetylation of GATA-3 regulates its DNA binding capacity and target gene expression [2]. Consequently, HDAC inhibition impairs GATA-3 dependent transcription [43]. Both GATA-3 and CDK9 are post-transcriptionally regulated by eIF4E, as intranuclear eIF4E binding is required for their XPO-1 dependent nuclear export [45]. Collectively, these findings suggest that HDAC and selective XPO-1 inhibition may warrant future study in combination with CDK9 inhibition as novel strategies targeting GATA-3 and the GATA-3 dependent transcriptome.

GATA-1, a GATA family member with an important role in megakaryocyte and erythrocyte development, binds Cyclin T1 and promotes CDK9 activation [57]. Therefore, the work presented here may suggest a similar mechanism by which GATA-3 directly binds and recruits (or stabilizes) the CDK9/Cyclin T1 complex, promoting efficient transcriptional elongation at its target genes. It is notable that transcription of a subset of GATA-3 target genes was significantly impaired upon CDK9 inhibition. This may be explained, at least in part, by a relatively long half-life for transcripts that were less affected by CDK9 antagonism. Therefore, the relatively short (6 h) exposure time we utilized may underestimate the true impact of CDK9 inhibition on the GATA-3 dependent transcriptome. This assertion may be supported by the observation that significant transcriptional pausing upon CDK9 inhibition was observed at most (70%) GATA-3 target genes. However, we cannot exclude alternative mechanisms, as GATA-3 may displace or cooperate with other CDK9/Cyclin T1 binding partners, including other transcription factors [57, 79, 80] or HEXIM, which sequesters and inactivates CDK9 within a larger 7SK snRNP complex [81–85].

As GATA-3’s role in T-cell lymphomagenesis is best explained by the transcriptional program (i.e., its target genes) it instigates, the work presented here interrogating the role of CDK9 in transcriptional regulation was largely reliant on integrated RNAP2 ChIP-seq and RNA-seq approaches. Consequently, the epigenetic effects of CDK9 inhibition, while incompletely understood, were not examined, but are relevant in a T-cell lymphoma context [86]. Components of the SWI/SNF complex are recurrently altered in many T-cell lymphomas [11, 87], and *SMARCA4* (BRG1) is dephosphorylated upon CDK9 inhibition, contributing to reactivation of gene expression [86].

In summary, we have shown that CDK9 is a dependency and therapeutic vulnerability across diverse T-cell lymphoma subsets, including those that are MCL-1 dependent. Among those that are MCL-1 independent, we have identified both GATA-3 dependent and independent transcriptional programs and biological processes (Fig. 5P) that are reliant on CDK9 activation. Therefore, CDK9 antagonists had significant activity across diverse, including genetically high-risk (e.g., p53 deficient), and orthogonal T-cell lymphoma specimens and model systems. Given the clinical development of selective CDK9 kinase inhibitors and degraders [39], these findings have significant therapeutic implications for this group of lymphomas for which novel therapeutic strategies are needed.

## Supplementary information

Supplemental methods, Table S1-S3 and Figures S1-S4

## Data Availability

All ChIP-seq and RNA-seq data will be available at GEO under accession number GSE296610. Public data were obtained from GSE36172 and GSE160119. Additional details about materials and methods are provided in the Supplemental Methods.
