# Supplementary material for: CDK9 is a dependency in GATA-3 driven and MCL-1 independent T-cell Lymphomas
Source: Blood Cancer J. 2025 Nov 27;16(1):9. doi: 10.1038/s41408-025-01427-1 (PMC12783670; doi:10.1038/s41408-025-01427-1)
Supplement: Supplementary file 1 — Supplemental methods, Table S1-S3 and Figures S1-S4 [file 41408_2025_1427_MOESM1_ESM.pdf]

# CDK9 is a Dependency in GATA-3 driven and MCL-1 independent T-cell Lymphomas

Chenguang Wang[1**†], Suhaib Abdelrahman[1†], Xiangrong Geng[1†], Alyssa Burgess[2], Ying S Hu[2], Mohd Ahmar Rauf[1], Nermin Kady[1,3], Yao Fu[1], Tara A. Reilly[1], Ira P. Maine[1], Phillip Boonstra[4,] Kirill Sabitov[4], Carlos Murga-Zamalloa[5], Ryan A. Wilcox[1**]

[1]Department of Internal Medicine, Division of Hematology and Oncology, University of Michigan, Ann Arbor, Michigan; [2]Department of Chemistry, College of Liberal Arts and Sciences, University of Illinois Chicago, Chicago, Illinois; [3]Clinical Pathology Department, Faculty of Medicine, Mansoura University, Mansoura, Egypt; [4]Department of Biostatistics, School of Public Health, University of Michigan, Ann Arbor, Michigan; [5]Department of Pathology, University of Illinois Chicago, Chicago, Illinois

**Correspondence to:

Chenguang Wang, University of Michigan Rogel Cancer Center, 4310 Cancer Center, 1500 East Medical Center Drive, Ann Arbor, MI 48109; phone: 734-355-3978; fax: 734-647-9654; e-mail: wchengua@med.umich.edu.

Ryan A. Wilcox, MD, PhD, University of Michigan Rogel Cancer Center, 4310 Cancer Center, 1500 East Medical Center Drive, Ann Arbor, MI 48109; phone: 734-615-9799; fax: 734-647-9654; e-mail: rywilcox@med.umich.edu.

†Contributed equally

**Running title: CDK9 in T-cell lymphomas**

**Keywords: CDK9, GATA-3, Ribosome Biogenesis, MCL-1, PTCL**

**Text word count: 3935**

**Figures: 5**

## Supplemental Methods

### Patients and cell lines

Cell lines were obtained from either American Type Culture Collection (ATCC) or Leibniz Institute (DSMZ), as previously described (1-3). T8ML-1 cells were kindly provided by Drs. Fujiwara and Yasukawa, and were cultured as previously described (4). All cell lines were mycoplasma free and independently authenticated by short tandem repeat (STR) profiling, performed by ATCC, and immunophenotyping (data not shown). Primary malignant T cells for *ex vivo* studies were isolated from patients with Sezary syndrome, as previously described (2, 4). Most cell lines are p53-deficient and have been genetically characterized, as previously described (3). Patient-derived xenograft models (PDX) models were generated by implanting 5-15 $\times 10^6$ patient-derived malignant T cells subcutaneously into NSG mice. Upon tumor engraftment, mice were treated with AZD4573 (or vehicle control) twice weekly for 2-3 weeks, as described further below. Explanted tumor cells were isolated for downstream analysis. Cells were cultured in RPMI 1640 supplemented with 10% FBS, 1 mM L-glutamine, and 100 µg/mL of penicillin and streptomycin at 37°C in a humidified atmosphere with 5% $CO_2$.

### Immunohistochemistry

All slides of lymphoma tissue sections were deparaffinized and stained on BOND RX autostainer (Leica Microsystems). For single chromogen XPO-1 staining, slides were subjected to antigen retrieval with BOND Epitope Retrieval solutions ER1 (Leica Microsystem, #AR9961) for 40 min at 99°C and stained using BOND Polymer Refine Detection kit (Leica Microsystems, #DS9800). The non-specific signal was blocked by incubating the slides with peroxide block for 15 minutes and protein block (Background Sniper, Biocare Medical, #BS966) for 15 minutes at room temperature. Slides were stained with Rabbit anti-CDK9 monoclonal antibody (1:300) for 30 min, and the signal was detected with polymer-HRP/DAB. PTCL cases were independently reviewed by hematopathologists (C.M.Z.).

### Genetically engineered and patient-derived xenograft mouse models

Mouse studies were approved by the University Committee on Care and Use of Animals (UCUCA) and performed in accordance with guidelines established by the Unit for Laboratory Animal Medicine (ULAM) at University of Michigan. Mice were housed under specific-pathogen free conditions. Treatment allocation was randomized, and all animals in given experiments were included for analysis. Floxed (SNF5, PTEN, TP53) and CD4-Cre mice were obtained from Jackson Laboratory and were crossed). All GEM models used here have been previously described (1, 3, 5). Lymphoma development for the experiments described was determined by the development of massive hepatosplenomegaly and/or bulky lymphadenopathy (>5mm). For adoptive transfer experiment, $5\times 10^6$ bulk splenocytes obtained from lymphoma-bearing donor mice were retro-orbitally injected into 12-16 weeks old recipient C67BL/6J mice. For short-term experiments, recipient C57BL/6J mice were monitored after adoptive transfer of splenocytes and upon lymphoma engraftment (demonstrated by palpable splenomegaly, usually within 2-4 weeks following adoptive transfer), mice were dosed with vehicle or AZD4573 by intraperitoneal injection. AZD4573 (generously provided by AstraZeneca) was dosed at 15 mg/kg, twice daily for 2 consecutive days with two hours interval for 1-3 weeks. For short-term treatment experiments, tumor burden was evaluated by organ weights and the extent of CD3, TCR-Vβ and Ki67 expression in splenocytes were determined by flow cytometry. T8-28 TCL model was also used for short-term AZD4573 experiments. Recipient BALB/c mice were monitored after

adoptive transfer of splenocytes from T8-28 lymphoma bearing mice. For PDX models, a core-needle biopsy or PBMC from PTCL, NOS specimen was blunt dissected and resuspended in 100 μL Matrigel and injected in the shaved flank of an NSG mouse (Jackson laboratory). For passage, tumors were mechanically dissociated and 5-10x$10^6$ cells were similarly injected in Matrigel subcutaneously. The PDX models established were utilized for experiments at passages 2-27. Prior to experiments, the fidelity of each PDX model was confirmed by histopathology in comparison to the original diagnostic biopsy specimen. Outcomes for PDX models were reported in accordance with recently published NCI PDXNet consensus guidelines (6).

**Patient-derived xenograft experiments analysis**

Longitudinal tumor volume trajectories were modeled using a generalized linear mixed model (GLMM) with a Gamma distribution and log link to account for the non-negative, right-skewed outcome. Let $TV_{ijk}$ denote the $k$-th tumor volume measurement (in mm$^3$) for mouse $j$ from PDX model $i$, which is observed on day $t_{ijk}$. Each $TV_{ijk}$ value was incremented by 1 so that resulting values were strictly positive in the model.

Let $\tau_i^{(r)}$ denote the day of the $r$-th AZD4573 treatment administration, which was shared for all treated mice within the same PDX model. We defined the following time variables for modeling purposes:

$$t_{ijk}^{baseline} \coloneqq t_{ijk} - t_{ij1},$$

$$\left(t_{ijk} - \tau_i^{(r)}\right)_+ \coloneqq \mathbb{I}\left(Group_{ij} = Rx\right) \cdot \max\left(0, t_{ijk} - \tau_i^{(r)}\right), \quad r = 1,2,3$$

Here, $t_{ijk}^{baseline}$ is the number of days elapsed since the first tumor volume measurement, and $\left(t_{ijk} - \tau_i^{(r)}\right)_+$ is the number of days since the $r$-th AZD4573 treatment administration. In this study, mice from PDX16 received one AZD4573 treatment; those from PDX15, PDX24, and PDX26 received two treatments, and mice from PDX10 received three treatments.

We used Akaike Information Criteria (AIC) to guide the specification of the mean model structure. The selected model was:

$$\begin{aligned} \ln\left(\mathbb{E}\left[TV_{ijk} \mid \cdot\,\right]\right) = \beta_1 \ln\left(TV_{ij1}\right) + \beta_2 t_{ijk}^{baseline} \\ + \beta_3 \left(t_{ijk} - \tau_i^{(1)}\right)_+ \\ + \beta_4 \left(t_{ijk} - \tau_i^{(2)}\right)_+ \\ + b_{1ij} t_{ijk}^{baseline} + b_{1i} t_{ijk}^{baseline}, \end{aligned}$$

where $b_{1ij}$ and $b_{1i}$ are nested mouse- and PDX-level random slopes, respectively.

Under this model, tumors in the control group (and in the AZD4573 treatment group prior to treatment) are assumed to have constant log-linear growth governed by $\beta_2$. Upon initial AZD4573 treatment, this growth trajectory is shifted by $\beta_3$ and further shifted by $\beta_4$ having received a second AZD4573 treatment. We did not incorporate any potential treatment effects for a third AZD4573 treatment due to small sample size and lack of statistical information for estimating such an effect.

Of primary interest are $\beta_3$ and $\beta_4$, which represent the marginal effects of initial and subsequent AZD4573 treatments over time, respectively.

Without treatment, tumors grew at an estimated 0.116 log-mm$^3$ (95% CI: [0.043, 0.189]; $p = 0.002$) per day, corresponding to 12.2% day-over-day growth. Upon receiving one AZD4573 treatment, growth decreased by -0.281 log-mm$^3$ per day (95% CI: [-0.339, -0.212]; $p < 0.001$) relative to controls, translating to an overall estimated decrease in tumor volume of 0.159 log-mm$^3$ per day after initial treatment, or about 14.7% day-over-day shrinkage. Following a second round of AZD4573 treatment, tumors exhibited a further slowing in their growth rate relative to those treated with one round, corresponding to an additional reduction of 0.038 log-mm$^3$ per day. However, this effect was not found to be statistically significant ($p = 0.426$). Predictions were generated under a standardized hypothetical scenario in which all mice were observed for 28 days prior to treatment, with baseline tumor volume fixed at 1000 mm$^3$ on day 27. We simulated the projected effects of receiving 0, 1, or 2 AZD4573 treatments, administered at 7-day intervals (on days 28 and 35), and tracked tumor progression for 14 days following initial treatment.

**Bioinformatics analysis of single cell RNA sequencing**

Single-cell RNA database was used for gene enrichment analysis, as previously described (1). Briefly, data from single patient was integrated using the concatenate function in anndata. Scrublet (v0.2.3) was applied to obtain per-cell scrublet scores and used for doublet removal. Cells with greater than 30% mitochondrial gene expression or expression of fewer than 200 detected genes were excluded. Genes that were expressed in fewer than 3 cells were also removed. Data normalization and highly variable genes were processed by Scanpy (v1.9.6) (7). Batch correction and integration were performed using scVI (1.2.2) (7). Normal skin cell datasets were also integrated to identify malignant T cells by inferring copy number variation (CNV) based on scRNA-seq data using InferCNV (v1.18.1). Neighborhood was calculated and embedded using the umap function. The neighborhood graph was clustered using leiden function in Scanpy. Annotation was performed based on well-established and lineage-specific cell markers or Celltypist (v1.6.3) (8).

**Cell viability**

Cell viability was analyzed by CellTiter-Glo® MT Cell Viability Assay (Promega #G9241) according to manufacturer's instructions. Briefly, equal volumes of cells and reagents were mixed and seeded to 96-well plate in triplicate or quadruplicate. Luminescence was measured after 10 min incubation. Data shown was performed in at least 3 independent experiments.

**RNA isolation, sequencing and analysis**

RNAs were extracted using RNeasy Mini Kit (Qiagen #74106), and on column DNase I digestion (Qiagen, RNase-free DNase set #79254) was performed during extraction to avoid DNA contamination. RNA concentration was determined by NanoDrop™ One/Onec Spectrophotometer (Thermo Fisher Scientific). Samples were prepared using the NEBNext Ultra II Directional RNA Library Prep Kit for Illumina (NEB #E7760L), Ribo depletion Module NEBNext rRNA Human/Mouse/Rat (NEB #E6310X) and NEBNext Multiplex Oligos for Illumina Unique dual (NEB #E6440L), where 100 ng of total RNA was ribosomal depleted using the rRNA Depletion module. The rRNA-depleted RNA is then fragmented 7 minutes determined by RIN (RNA Integrity Number) of input RNA as per protocol, and copied into first strand cDNA using reverse transcriptase and dUTP mix. Samples undergo end repair and dA-Tailing step followed by ligation of adapters. The products are purified and enriched by PCR to create the final cDNA

library. Final libraries were checked for quality and quantity by TapeStation (Agilent) and Qubit (Thermo Fisher Scientific). This pool was subjected to 151bp paired-end sequencing according to the manufacturer's protocol (Illumina NovaSeq). Bcl2fastq2 Conversion Software (Illumina) was used to generate de-multiplexed Fastq files. For quantitative RT-PCR, complementary DNA (cDNA) was prepared using the QuantiTect Reverse Transcription Kit (Qiagen #205311). qPCR was performed using the Radiant SYBR Green H-ROX qPCR Kits (Alkali Scientific Inc. #QS2020) and analyzed using the ΔΔCt method with control samples set as 1. Primers used in this study were in Supplemental Table 2.

**Gene expression analysis**

Gene expression analysis was performed as previously described (GATA3 paper). Briefly, raw reads were quality checked with FastQC (v0.11.8) and adapter trimmed with Cutadapt (v2.3). Trimmed reads were mapped to the reference genome GRCh38 (ENSEMBL) using STAR (v2.7.8a) and assigned count estimates to genes using RSEM (v1.3.3) with default parameters. Differentially expressed genes were determined using expected counts by DESeq2 package (v1.32), p-value <-0.05 and fold-change >=1.5 were considered statistically significant. Heatmap were generated by pheatmap (v1.0.12) using 'ward.D' clustering method. Pathway enrichment analysis was performed by Metascape (v3.0) or gene set enrichment analysis (GSEA) with default parameters. Activity Scores were calculated using Hallmark gene sets by Gene Set Variation Analysis (GSVA, v1.42.0). Public datasets (GEO Accession number: GSE36172 and GSE160119) were also collected for plotting.

**Immunoblotting (IB)**

Cells were lysed in RIPA buffer (Thermo Fisher Scientific, #89900) containing protease inhibitor. After centrifuging at 13,000 x g. for 10 min at 4°C, the protein concentration of the supernatant was quantified by Pierce BCA protein assay. 30~40 µg cell lysates were boiled in SDS gel sample buffer at 70 °C for 10 min. Samples were run using 4%-12% NuPAGE Bis-Tris protein gels and transferred to PVDF membranes (Bio-Rad #1620177). Primary antibodies were incubated overnight, and horseradish peroxidase (HRP) conjugated anti-mouse or anti-rabbit IgG antibodies were used at 1:1000. Blots were developed using SuperSignal West Femto (Thermo Fisher Scientific #34096). Primary antibodies used are provided in Supplemental Table 3.

**BH3 profiling**

BH3 profiling was performed using JC-1 plate based or cytochrome c based intracellular BH3 (iBH3) procedures, as previously described (9). For the JC-1 method, cells were washed with PBS once, then 2 x $10^4$ cells/well were dissolved in 15 µL MEB buffer (150 mM mannitol, 10 mM HEPES-KOH pH 7.5, 50 mM KCl, 0.02 mM EGTA, 0.02 mM EDTA, 0.1% BSA, and 5 mM succinate). 2 x dye solution containing 20 µg/mL oligomycin, 50 µg/mL digitonin, 2 µM JC-1 and 10mM ß-mercaptoethanol in MEB was freshly prepared. 2 x Peptides and 2 x controls (2% DMSO and 10 µM FCCP) were prepared in 2 x dye and then 15 µL/well was added to black, flat bottom 384-well plates (Corning 3573). 2 x $10^4$ cells were then loaded into the same plate in technical triplicates. A total of 15 µL cells and 15 µL 2 x Peptides / controls stood at room temperature for 10 minutes to allow permeabilization. Final peptides concentrations used were BIM at 0.5, 1, and 5 µM, PUMA at 5 and 10 µM, BAD at 5 and 10 µM, HRK at 5, 10 and 80 µM, MS1 and FS1 at 5 and 10 µM. Peptides were purchased from GenScript and stored at -80°C as 1~10 mM solution in DMSO.

Plates were analyzed on a BioTek Synergy HT plate reader at an excitation of 545 nm and emission of 590 nm every 10 minutes for 3 hours at 30°C. The resulting relative fluorescent units were plotted versus time and area under the curve (AUC) values were generated using GraphPad Prism9 software. 1% DMSO and 5 μM FCCP were used to set 0% and 100% depolarization parameters, respectively. The following equation was used to generate normalized values for treatment samples:

$$\%\ \mathrm{mitochondrial\ depolarization} = 100 - \left[100\ \mathrm{x}\ \left(\frac{\mathrm{AUC_{Sample}} - \mathrm{AUC_{FCCP}}}{\mathrm{AUC_{DMSO}} - \mathrm{AUC_{FCCP}}}\right)\right]$$

For the iBH3, 5 x $10^5$ cells were resuspended in 100 μL MEB buffer (150 mM mannitol, 10 mM HEPES-KOH pH 7.5, 150 mM KCl, 1 mM EGTA, 1 mM EDTA, 0.1% BSA, and 5 mM succinate) in each FACS tube. Then 100 uL of 0.002% (w/v) digitonin in MEB with 2 x Peptides or 2 x controls ( 2% DMSO and 50 μM Alamethicin Ala) were added into same tube and incubated for 60 min at room temperature. Cells were fixed with 67 uL of 4% formaldehyde for 10 min. Fixation was terminated by adding 67 uL of N2 (1.7M Tris, 1.25M Glycine pH 9.1) for 5 min. Then cells were stained with 40 uL /tube of 10 x CytoC Stain Buffer (10% BSA, 2% Tween20, PBS, filtered) with a 1:40 dilution (BD Biosciences, anti-cytochrome c Alexa Fluor 647) for 60 - 90 min under the dark. Flow cytometry was used to measure the median fluorescence intensity (MFI) of cytochrome c retained in the mitochondria. 1% DMSO and 25 μM Ala were used to set fully retention and complete loss of cytochrome c, respectively. The following equation was used to calculate released cytochrome c under each peptide:

$$\%\ \mathrm{cytochroma\ c\ loss}\ =\ 1 - \left(\frac{\mathrm{MFI_{Sample}} - \mathrm{MFI_{Ala}}}{\mathrm{MFI_{DMSO}} - \mathrm{MFI_{Ala}}}\right)$$

### Microscopy

Eight-well chambered cover glasses (Cellvis, cat#C8-1-N) were coated with 200 μL of 0.01% PLL solution (Milipore Sigma, cat#P4707) and incubated for 2 hours at 37 °C. Prior to cell landing, the chambered cover glass was pre-warmed, and the solution was aspirated. Cells were immobilized onto a poly-L-lysine (PLL)-coated cover glass by incubating the cells in sterile DPBS for 30 min at 37 °C and 5% $CO_2$. Cells were manipulated gently, and the subsequent solutions were introduced slowly into the corners of the wells to minimize cell detachment from the glass surface. Following the incubation period, cells were fixed with 4% paraformaldehyde in DPBS for 15 minutes at room temperature. Subsequently, cells were blocked and permeabilized in a blocking buffer with 5% BSA and 0.3% Triton X-100 in DPBS for 90 min. Immunostaining was performed using FBL antibody (1:200 dilution) and L7a antibody (1:60) in 1% BSA and 0.3% Triton X-100 in DPBS overnight at 4 °C. Cells were subsequently incubated with secondary antibody, goat anti-rabbit Superclonal Alexa Fluor™ 647 (5 μg/mL) or Alexa Fluor™ 488- conjugated goat anti-mouse secondary antibody (5ug/mL) in 1% BSA and 0.3% Triton X-100 for 2 hours at room temperature. Following the staining, the samples were washed three times with DPBS for 5 min each. DAPI stain was added to each well and allowed to incubate for 10 min prior to imaging. For L7a staining, widefield images were captured using a Nikon Ti-2 Eclipse inverted microscope equipped with a CrestOptics DeepSIM imaging system, Nikon 20x dry objective with DeepSIM mask moved out of the optical path. Imaging was performed with a Kinetix sCMOS camera (serial number: A23H723002). NIS-Elements Advanced Research Software (version: 5.42.04) was used for image acquisition. Laser lines of 405 nm and 640 nm

were employed, with camera exposure times set to 50 ms and 100 ms, respectively. Super-resolution imaging was acquired using the DeepSIM system with a Nikon 60x/1.42 oil immersion objective. Volumetric images were captured using the Deep imaging mode with an integration time of 50 ms and 100 ms in the 405 and 647 channels, respectively, with a step size of 0.5 μm. Image volumes captured with DeepSIM were reconstructed within NIS-Elements Software (5.42.04), followed by blind 3D Deconvolution. Blending: Alpha. From the widefield images, a minimum of 10 regions of interest (ROIs) were selected for each treatment and DMSO condition. The mean number of cells exhibiting the L7a positive phenotype in each ROI was determined by analysis with ImageJ, then plotted. For FBL staining, Widefield images were captured using a Nikon Ti-2 Eclipse inverted microscope equipped with a Nikon Intensilight C-HGFI light source, and Nikon 40x/1.30 oil immersion objective. Imaging was performed with a Prime 95B sCMOS camera (Serial Number: A18B203004). NIS-Elements Advanced Research Software (version: 5.42.04) was used for image acquisition. Nikon Fluorescence Filter cubes ET DAPI (96360) and ET GFP (96362) were employed, with camera exposure set to 80 ms and 50 ms, respectively at 12.5% transmittance (ND 8). All imaging was performed at room temperature. Image analysis was performed using ImageJ, with segmentation carried out via the Trainable Weka Segmentation plugin (version 3.3.2). A pixel classifier was trained to identify nucleolar regions while excluding non-specific staining and background. The trained classifier was saved and uniformly applied to all regions of interest (ROIs) within each replicate experiment: consisting of two treatment conditions (DMSO and AZD4573), with a minimum of 10 ROIs analyzed per condition, per replicate (n=2 replicates). The output probability maps were thresholded to generate binary masks, with background pixels set to NaN to exclude them from further analysis. Nucleolar regions were identified using the Analyze Particles function in ImageJ, with parameters set to obtain area measurements. Identified nucleoli were manually grouped by cell and the median nucleolar area was calculated per cell. These per-cell median values were pooled across both replicates for statistical comparison (n=125, per condition).

### Chromatin immunoprecipitation (ChIP) and sequencing

ChIP was performed using SimpleChIP® Plus Enzymatic Chromatin IP Kit (Cell Signaling #9004) with minor modifications. Briefly, 5-10 million cells per IP were fixed using formaldehyde (1% final concentration) for 10 min at room temperature and were stopped with 125 mM glycine addition for 5 min at room temperature. After washing, cells were lysed in prepared Buffer A for 10 min at room temperature. After lysing, nuclei were collected (500 × g, 4°C, 3 min) and digested with prepared Buffer B and Micrococcal Nuclease for 20 min at 37 °C. After adding 50 mM EDTA to stop digestion, nuclei were collected (16,000× g, 4°C, 1 min) and resuspend in prepared ChIP buffer. Nuclei were then sonicated with two pulses of 40 seconds each and 30 s incubation on wet ice at setting 4 using an ultrasonic cell disruptor (Microson) with 18-inch probe. Chromatin was clarified (16,000× g, 4°C, 10 min) and incubated with primary antibody and 0.4 μg IgG antibody overnight. Chromatin was then incubated with 30 μL Dynabeads Protein G (Invitrogen #10004D) for 4 hours and washed with low-salt washing buffer three times and high-salt washing buffer once. Elution and de-crosslinking were performed overnight in 150 elution buffer (300 mM NaCl, 5 mM DTT and 0.1% SDS in TE buffer, pH8.0) at 65 °C, and RNA and proteins were digested by adding RNase A and proteinase K, respectively. Eluted samples were purified by QIAquick™ PCR Purification Kit (Qiagen #28104) and were ready for high-throughput sequencing. For ChIP-seq library preparation, NEBNext ChIP-Seq Library Prep Master Mix Set for Illumina (NEB #E6240) was used according to the manual’s instructions.

Library quality was determined by TapeStation prior to being sequenced on the Illumina NovaSeq X plus platform.

### ChIP-sequencing analysis

ChIP-seq analysis were performed as previously described (3). Briefly, raw reads were mapped to reference genome hg38 (UCSC) using bwa (v0.7.15) and PCR duplicates and multimapping reads were marked using Picard MarkDuplicates (v2.26.5). Peak calling was performed by MACS2 (v2.1.2) to generate bed files with following parameters (other parameters were used as default): -f BAM --keep-dup auto -p 0.01 -B. Peak annotation was performed by ChIPseeker (v1.45.0). Heatmaps and profile plot for various ChIP-seq were created by Deeptools (v2.0). Traveling ratios were determined by the ratio of RNA Pol II bound at the promoter (ChIP-seq signal ±300 bp of the TSS) to RNA Pol II bound within the gene-body (ChIP-seq signal spanning 300 bp downstream of the TSS to 3 kb downstream of the gene end). Other plots were generated in-house R scripts. For rDNA mapping, raw reads were mapped to custom hg38 genome that contained rDNA sequence (NCBI Genbank KY962518.1) using Bowtie2 with following parameters: -X 2000. Samtools view -q 1 was used to allow for multimapping reads (10). Single base resolution genomic track on rDNA were analyzed using deeptools to generate read density matrix at every nucleotide in the rDNA sequence. The read density was normalized to the median signal across the rDNA sequence for that dataset, thereby setting median coverage across each rDNA track to 1.

### 47S-FISH

FISH was performed as previously described (11, 12). Briefly, Fluorescent FISH probes specific to 47S pre-rRNA obtained from previous publications and custom synthesized conjugated with Cy5 (IDT). The pool of FISH probe set (5 nmol) was reconstituted in TE to achieve 12.5 uM stock. 5 x $10^6$ cells were harvested and washed twice with 750 µl of PBS containing 2 mM EDTA. Pelleted cells were resuspended in 750 µl of 3.7 % formaldehyde, and incubated at 37 °C for 10 min with gentle shaking. At time of staining, 2.5 x $10^5$ cells were transferred to a 96 well plate and washed using 150 µl of FISH wash buffer (10 % formamide in 2X saline-sodium citrate (SSC) buffer [300 mM Sodium Chloride, 30 mM Sodium citrate pH 7.0]). After washing, cells were resuspended in 50 µl of Hybridization buffer (10 % Dextran sulfate, 10 % formamide in 2X SSC) containing 0.5 µM fluorescent probe pool mix. Plates were sealed using parafilm and covered with aluminum foil and incubated at 37 °C overnight. After incubation, cells were pelleted and resuspended for washing in 150 µl of FISH wash buffer, and then re-pelleted and resuspended in 150 µl of FISH wash buffer for incubation at 37 °C for 30 min. Following incubation, cells were pelleted, resuspended in 150 µl of 4′,6-diamidino -2-phenylindole [DAPI] (100 ng/ml prepared in FISH wash buffer), and incubated at 37 °C for 30 min. Finally, cells were washed in 150 µl 2X SSC buffer, and resuspended in 150 µl of 2X SSC plus 150 µl of 1X PBS. 47S-FISH and DAPI intensity per cell were acquired Attune Cytpix Flow Cytometer (ThermoFisher) and the data were analyzed by FlowJo (BD Bioscience).

### Flow Cytometry

Commercially available fluorochrome-conjugated antibodies were utilized for flow cytometry (Supplementary Table 2). For intracellular (Ki67) staining, cells were fixed and permeabilized in Foxp3 Fix/Perm buffer (Biolegend cat# 421403) and then stained with appropriate antibodies. Flow cytometry data was acquired using Attune Cytpix Flow Cytometer (ThermoFisher) and the data were analyzed by FlowJo (BD Bioscience).

**Dual-luciferase assay**

The GATA-3 promoter (-148 bp to +587 bp), previously published (13), was cloned into pGL4.10 vector (Promega). HEK293T cells were grown in 24-well plates and transfected with 0.2 µg pGL4.0-*GATA-3*-luc and 0.02 µg *Renilla* luciferase vector (pGL4.70; Promega), together with 0.2 µg pLVX-AcGFP (control) or 0.2 µg pLVX-*AcGFP GATA-3* or/and 0.6 µg Flag-p300 plasmid as indicated. 48 hours after transfection, transfected HEK293T cells were harvested in Glo lysis buffer (Promega #E266A) and luciferase assays were performed using Dual-Luciferase reporter assay system (Promega #E2940). Firefly value was normalized to *Renilla* value. The relative luciferase activities (RLUs) were expressed as a percent of the luciferase activity in every group vs. the pLVX-AcGFP plasmid transfection-induced luciferase activity. Three independent experiments were performed.

**Supplemental Table 1. Summary of cell lines and primary cells used in this study showing their disease subtype, GATA-3 status, MCL dependence and IC50 for MCL1 CDK9 selective inhibitor.**

| Cell line | Disease | GATA-3 | MCL dependence | IC50 (nM) | |
|---|---|---|---|---|---|
| | | | | S63845 | AZD4573 |
| H9 | CTCL | + | Independent | >800 | 96.09 |
| MyLa CD4 | CTCL | + | Independent | >800 | 543.4 |
| MOLT4 | T-ALL | + | Independent | >800 | 39.84 |
| CCRF-CEM | T-ALL | + | Independent | >800 | 18.52 |
| SUP-T1 | T-ALL | + | Independent | >800 | 51.55 |
| MAC1 | CTCL | + | Dependent | 53.74 | 7.022 |
| SUP-M2 | ALCL | + | Dependent | 508.8 | <1 |
| SUDHL1 | ALCL | + | Dependent | >800 | 13.34 |
| SR-786 | ALCL | + | Dependent | 356.8 | 9.99 |
| Karpas299 | ALCL | - | Dependent | >800 | 15.94 |
| DEL | ALCL | + | Dependent | 43.55 | 27.21 |
| T8ML1 | PTCL, NOS | + | Dependent | 1.3 | 6.651 |
| Jurkat | T-ALL | - | Dependent | 143.1 | 22.59 |
| Pt. B | CTCL (SS) | + | NA | NA | 21 |
| Pt. L | CTCL (SS) | + | NA | NA | 64.59 |

**Supplemental Table 2. Primers used in this study.**

| Name | Sequence | Application |
|---|---|---|
| Satellite (SATE) Primers | #4486 (CST) | ChIP-qPCR |
| 28S-F | ACCCGAAAGATGGTGAACTATG | |
| 28S-R | GGTCGGACGACCGATTTG | |
| 18S-F | CCCTATCAACTTTCGATGGTAGTC | |
| 18S-R | TTGGATGTGGTAGCCGTTTC | |
| ITK-F | GCCTCCTCGTTTTGTGAATTTT | |
| ITK-R | GATGTCAAAGGCCGAAACCA | |
| CCR4-F | CTCTGGCTTTTGTTCACTGCTGC | qRT-PCR |

| | | |
|---|---|---|
| CCR4-R | AGCCCACAGTATTGGCAGAGCA | |
| C-MYC-F | CACATCAGCACAACTACGCA | |
| C-MYC-R | GGTGCATTTTCGGTTGTTGC | |
| GAPDH-F | TCTGACTTCAACAGCGACAC | |
| GAPDH-R | TGTCATACCAGGAAATGAGCTT | |
| HPRT1-F | GTGAAAAGGACCCCACGAAG | |
| HPRT1-R | TCCAAACTCAACTTGAACTCTCA | |
| ITK-F | GCCCAAAGTGATGGAACTAGAA | |
| ITK-R | CAGTAGAAGCCAGTGTCGAATAG | |
| NOB1-F | GCACTCACATACCAGTTGGAAGC | |
| NOB1-R | GAGGTGTTTCTGGGTGCTGAATC | |
| DDX10-F | GAGTTTGTCCGTAAGAGAGCTGC | |
| DDX10-R | GGCAGTTCTACCTGCTCTGTG | |
| NOL6-F | CTACAGACCTGACAGTCAACGG | |
| NOL6-R | AGAGGCAGTGACATCAGCACA | |
| RRP1-F | ATGCGGATGGTCCTGAACG | |
| RRP1-R | GAAGTGGCTCTTCACACCGTT | |
| MCL1-F | CCAAGAAAGCTGCATCGAACC | |
| MCL1-R | CAGCACATTCCTGATGCCAC | |
| 47S-F | GGCGGTTTGAGTGAGACGAGA | |
| 47S-R | ACGTGCGCTCACCGAGAGCAG | |
| 18S-F | CGACGACCCATTCGAACGTC | |
| 18S-R | CTCTCCGGAATCGAACCCTG | |
| 28S-F | GAGCTCAGGGAGGACAGAAA | |
| 28S-R | AGGTCAGAAGGATCGTGAGG | |
| SNF5-F | CACCATGCCCCCACCTCCCCTACA | Genotyping |
| SNF5-R | CAGGAAAATGGATGCAACTAAGAT | |
| CD4-Cre-F | TTCCCAACCAACAAGAGCTC | |
| CD4-Cre-R | GGACCGACGATGAAGCATGT | |
| P53-F | GGTTAAACCCAGCTTGACCA | |
| P53-R | GGAGGCAGAGACAGTTGGAG | |
| PTEN-F | CAAGCACTCTGCGAACTGAG | |
| PTEN-R | AAGTTTTTGAAGGCAAGATGC | |
| GATA3-F | CAGTCTCTGGTATTGATCTGCTTCTT | |
| GATA3-R | GTGCAGCAGAGCAGGAAACTCTCAC | |
| Human_47S#1 | AGAGGACAGCGTGTCAGC/3Cy5Sp/ | 47S-FISH |
| Human_47S#2 | AACCTCTCCAGCGACAGG/3Cy5Sp/ | |
| Human_47S#3 | CCGCGCGCATCCGGAGGC/3Cy5Sp/ | |
| Human_47S#4 | GTCACCGGTAGGCCAGAG/3Cy5Sp/ | |
| Human_47S#5 | AGGAGCGCGGCCGGCTAG/3Cy5Sp/ | |
| Human_47S#6 | CCCGGCAGGCGGCTCAAG/3Cy5Sp/ | |
| Human_47S#7 | GCGAGAGAACAGCAGGCC/3Cy5Sp/ | |

| | | |
|---|---|---|
| Human_47S#8 | GAGTCGGGACGCTCGGAC/3Cy5Sp/ | |
| Human_47S#9 | TGGGTCAGAGACCCGGAC/3Cy5Sp/ | |
| Human_47S#10 | CAGCGGAGAGCGCACGGG/3Cy5Sp/ | |
| Human_47S#11 | CGGGGTGGGGTTGTCGCG/3Cy5Sp/ | |
| Human_47S#12 | GACACGCACGGCACGGAG/3Cy5Sp/ | |
| Human_47S#13 | CCGCGGAGACGAGAACGC/3Cy5Sp/ | |
| Human_47S#14 | GGAAGGGGCGGCGGACAA/3Cy5Sp/ | |
| Human_47S#15 | CGGCCAGCGAGCCGATCG/3Cy5Sp/ | |

**Supplemental Table 3. Antibodies used in this study.**

| **Antigen** | **Clone(s)** | **Fluorochrome** | **Source (cat. #)** | **Additional information** |
|---|---|---|---|---|
| GATA-3 | Monoclonal | | Invitrogen #MA1-028 | IB: 1:2000<br>IP: 1-2 µg |
| Cleaved PARP | Monoclonal | | Cell Signaling #5625 | IB: 1:1000 |
| Cleaved Caspase3 | Monoclonal | | Cell Signaling #9664 | IB: 1:1000 |
| MCL-1 | Monoclonal | | Cell Signaling #5453 | IB: 1:1000 |
| RPB1 | Monoclonal | | Cell Signaling #2629 | IB: 1:1000<br>IP: 1:100 |
| pS2 | Monoclonal | | Abcam #238146 | IB: 1:1000<br>IP: 2 µg |
| CDK9 | Monoclonal | | Santa Cruz #sc-13130 | IP: 10 µg |
| CCNT1 | Monoclonal | | Cell Signaling #81464 | IP: 1:100 |
| p300 | Polyclonal | | Bethyl Laboratories #A300-358A | IP: 3-4 µg |
| RRP1 | Monoclonal | | Proteintech #14896-1-AP | IB: 1:500 |
| NOL6 | Monoclonal | | Proteintech #16361-1-AP | IB: 1:500 |
| NOB1 | Monoclonal | | Proteintech #10091-2-AP | IB: 1:500 |
| GAPDH | Monoclonal | | Cell Signaling #2118 | IB: 1:1000 |
| ITK | Monoclonal | | Cell Signaling #2380 | IB: 1:1000 |
| C-MYC | Monoclonal | | Cell Signaling #13987 | IB: 1:1000 |
| Rabbit IgG | Monoclonal | | Cell Signaling #7074 | IB: 1:1000 |
| Mouse IgG | Monoclonal | | Cell Signaling #7076 | IB: 1:1000 |
| CD3 | 145-2C11 | BV421 | BD biosciences #562600 | 0.2 µg |
| TCR-Vβ 8.1/8.2 | MR5-2 | FITC | BD biosciences #553185 | 0.5 µg |
| TCR-Vβ 2 | B20.6 | FITC | BD biosciences #553280 | 0.5 µg |
| TCR-Vβ 9 | MR10-2 | FITC | BD biosciences #510138 | 0.5 µg |
| Phospho-RPB1 | Monoclonal | | Invitrogen #MA5-32637 | 1:1000 |
| Rabbit IgG | Monoclonal | Alexa Fluor™ 647 | Invitrogen #A78957 | 2 µg/mL |
| RPL7a | Monoclonal | | Cell Signaling #2415S | 1:60 |
| Fibrillarin (FBL) | Monoclonal | | Proteintech #66985-1-Ig | 1:200 |

## References

1. Kady N, Abdelrahman S, Rauf A, Burgess A, Weiss J, Gunasekara H, et al. The GATA-3-dependent transcriptome and tumor microenvironment are regulated by eIF4E and XPO1 in T-cell lymphomas. Blood. 2024.
2. Geng X, Wang C, Abdelrahman S, Perera T, Saed B, Hu YS, et al. GATA-3 Dependent Gene Transcription is Impaired upon HDAC Inhibition. Clin Cancer Res. 2024.
3. Geng X, Wang C, Gao X, Chowdhury P, Weiss J, Villegas JA, et al. GATA-3 is a proto-oncogene in T-cell lymphoproliferative neoplasms. Blood cancer journal. 2022;12(11):149.
4. Wang T, Lu Y, Polk A, Chowdhury P, Zamalloa CM, Fujiwara H, et al. T-cell Receptor Signaling Activates an ITK/NF-kappaB/GATA-3 axis in T-cell Lymphomas Facilitating Resistance to Chemotherapy. Clin Cancer Res. 2017;23(10):2506-15.
5. Gao X, Wang C, Abdelrahman S, Kady N, Murga-Zamalloa C, Gann P, et al. Notch Signaling Promotes Mature T-Cell Lymphomagenesis. Cancer research. 2022;82(20):3763-73.
6. Meric-Bernstam F, Lloyd MW, Koc S, Evrard YA, McShane LM, Lewis MT, et al. Assessment of Patient-Derived Xenograft Growth and Antitumor Activity: The NCI PDXNet Consensus Recommendations. Mol Cancer Ther. 2024;23(7):924-38.
7. Virshup I, Bredikhin D, Heumos L, Palla G, Sturm G, Gayoso A, et al. The scverse project provides a computational ecosystem for single-cell omics data analysis. Nat Biotechnol. 2023;41(5):604-6.
8. Xu C, Prete M, Webb S, Jardine L, Stewart BJ, Hoo R, et al. Automatic cell-type harmonization and integration across Human Cell Atlas datasets. Cell. 2023;186(26):5876-91 e20.
9. Ryan J, Letai A. BH3 profiling in whole cells by fluorimeter or FACS. Methods. 2013;61(2):156-64.
10. George SS, Pimkin M, Paralkar VR. Construction and validation of customized genomes for human and mouse ribosomal DNA mapping. J Biol Chem. 2023;299(6):104766.
11. Antony C, George SS, Blum J, Somers P, Thorsheim CL, Wu-Corts DJ, et al. Control of ribosomal RNA synthesis by hematopoietic transcription factors. Molecular cell. 2022;82(20):3826-39 e9.
12. Antony C, Somers P, Gray EM, Pimkin M, Paralkar VR. FISH-Flow to quantify nascent and mature ribosomal RNA in mouse and human cells. STAR Protoc. 2023;4(3):102463.
13. Nakata Y, Brignier AC, Jin S, Shen Y, Rudnick SI, Sugita M, et al. c-Myb, Menin, GATA-3, and MLL form a dynamic transcription complex that plays a pivotal role in human T helper type 2 cell development. Blood. 2010;116(8):1280-90.

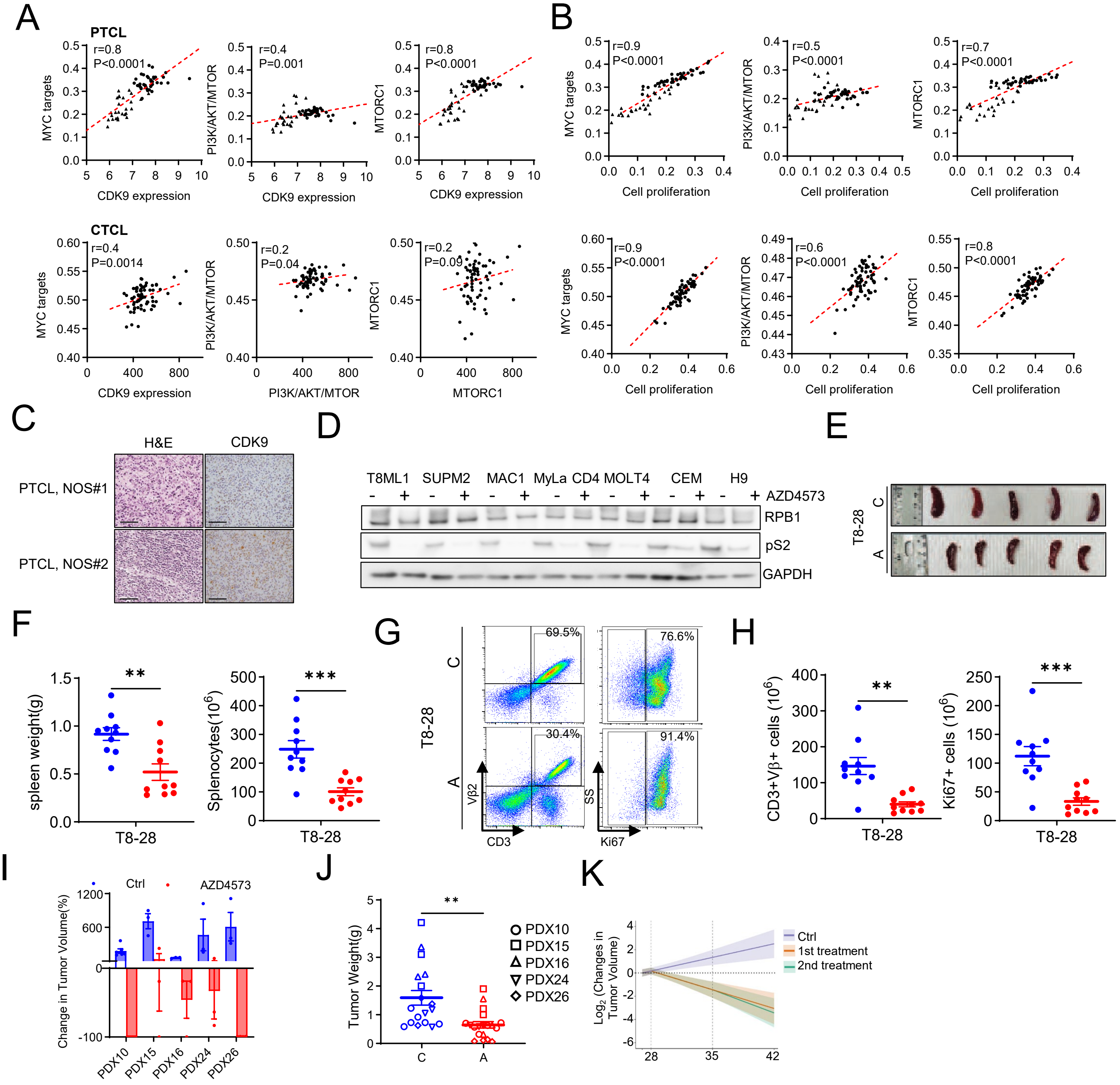

A
PTCL
r=0.8
P<0.0001
MYC targets
CDK9 expression
r=0.4
P=0.001
PI3K/AKT/MTOR
r=0.8
P<0.0001
MTORC1
CTCL
r=0.4
P=0.0014
r=0.2
P=0.04
r=0.2
P=0.09
B
r=0.9
P<0.0001
Cell proliferation
r=0.5
P<0.0001
r=0.7
P<0.0001
r=0.9
P<0.0001
r=0.6
P<0.0001
r=0.8
P<0.0001
C
H&E
CDK9
PTCL, NOS#1
PTCL, NOS#2
D
T8ML1 SUPM2 MAC1 MyLa CD4 MOLT4 CEM H9
AZD4573
RPB1
pS2
GAPDH
E
T8-28
C
A
F
spleen weight(g)
Splenocytes(10^6)
T8-28
G
69.5%
76.6%
30.4%
91.4%
Vβ2
CD3
SS
Ki67
H
CD3+Vβ+ cells (10^6)
Ki67+ cells (10^6)
I
Ctrl
AZD4573
Change in Tumor Volume(%)
PDX10
PDX15
PDX16
PDX24
PDX26
J
Tumor Weight(g)
PDX10
PDX15
PDX16
PDX24
PDX26
K
Log2 (Changes in Tumor Volume)
Ctrl
1st treatment
2nd treatment

**Supplemental Fig. S1. CDK9 is activated in TCLs.** (A) Correlation of enrichment score of MYC targets, PI3K/AKT/MTOR signaling and MTORC1 signaling and normalized CDK9 expression in public PTCL and CTCL (in-house) gene expression profiling datasets. (B) Correlation of enrichment score of MYC targets, PI3K/AKT/MTOR signaling and MTORC1 signaling and score of cell proliferation in public PTCL and CTCL (in-house) gene expression profiling datasets. (C) 19 PTCL cases were evaluated for CDK9 expression by immunohistochemistry. 17 cases were shown to be positive (cutoff: 20%). Representative photographs that express CDK9 within the tumor cells (right) and corresponding hematoxylin and eosin (H&E) stain (left) were shown. Scale bar is 60 uM. (D) RNA pol II (RPB1) and phospho-RNA pol II (pS2) IB was performed in multiple TCL cell lines with control and AZD4573 treatment (50 nM for 4 hours), including CTCL (H9, MyLa CD4 and MAC1), ALCL (SUP-M2), T-ALL (SUP-T1 and CEM) and PTCL, NOS (T8ML1). (E-F) Splenocytes from lymphoma-bearing T8-28 mice were adoptively transferred into syngeneic Balb/c recipients (n=5, 2 biologic replicates). Upon lymphoma engraftment, mice were treated with either vehicle control (C, blue circles) or AZD4573 (A, red circles). At the time of study termination, spleens were explanted and weighed. Representative examples are shown in E, and the data summarized in F. (G-H), Clonal T cells ($CD3^{+}V\beta^{+}$) in spleens were quantified and proliferation examined by Ki67 expression. Representative examples are shown in G, and the data summarized in H, as indicated. (I) Independent PTCL, NOS PDX (n=5) were established subcutaneously in NSG recipients (n=4-5/treatment group). Upon engraftment, PDX were randomized to treatment with vehicle control (blue) or AZD4573 (red), as before. Changes in tumor volume at baseline (first day of measurement) and at the end of treatment are shown in the waterfall plots. (J) On study termination, PDX were excised and weighed, and the data summarized. (K) Model-predicted population-level log2 fold change in tumor volume from baseline over time, stratified by AZD4573 treatment regimen. Shaded regions indicate 95% confidence bands. Vertical dashed lines on days 28 and 35 indicate treatment administration days. All regimens were initialized with a baseline tumor volume of 1000 $mm^3$ on day 27 under a hypothetical experimental scenario. Data are represented as mean ± SEM and were analyzed using Welch's unpaired t-test. Correlation was analyzed by two-tailed pearson correlation coefficients. **p<0.01, *** P<0.001.

A

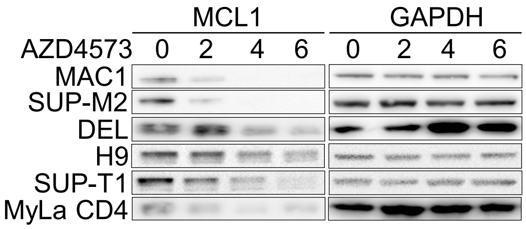


**Supplemental Fig. S2. CDK9 regulated MCL-1 in T cell lymphomas.** (A) MCL1 immunoblot (IB) was performed in TCL cell lines, including CTCL (H9, MyLa CD4 and MAC1), ALCL (SUP-M2 and DEL) and T-ALL (SUP-T1).

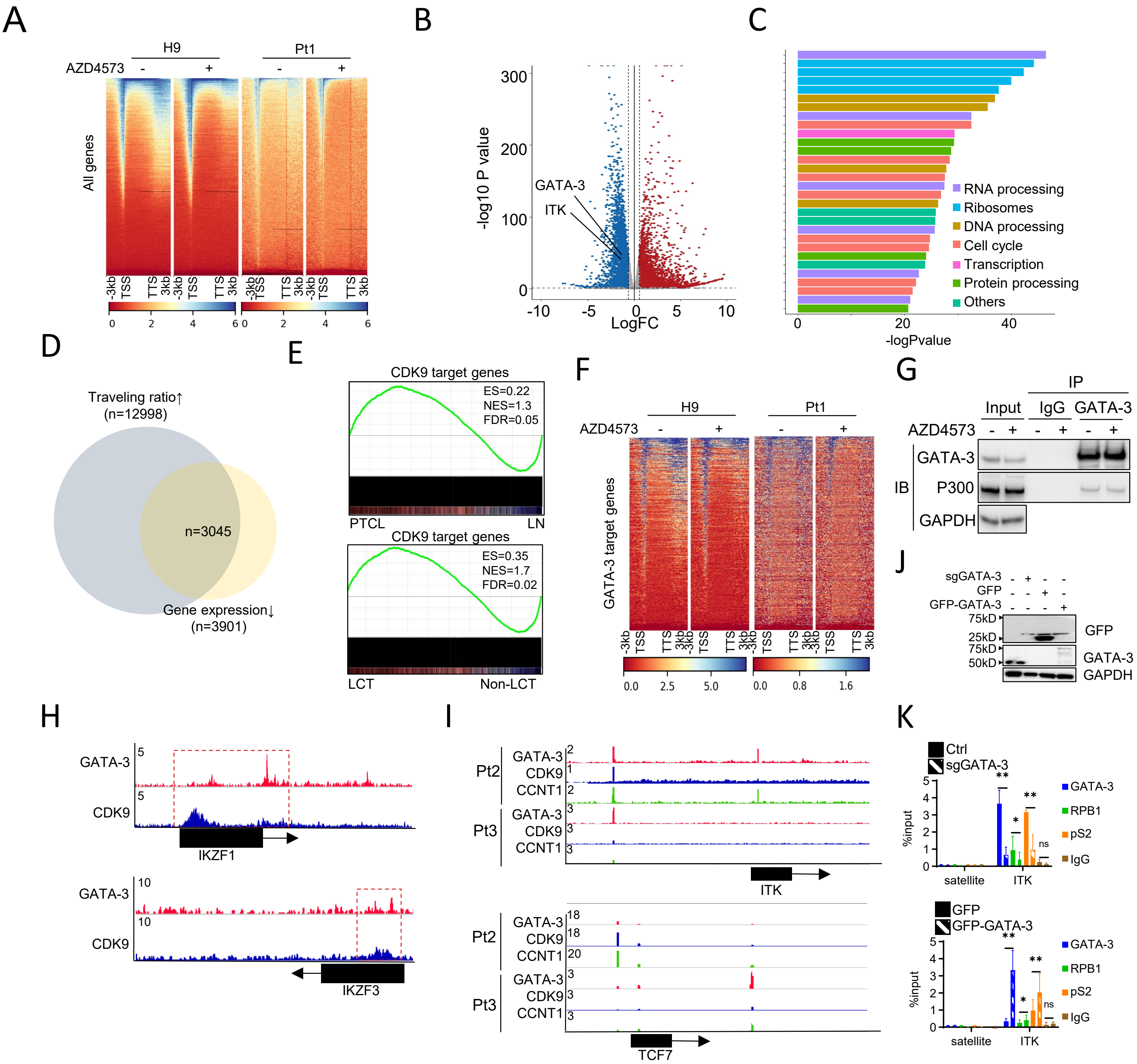

A
H9
Pt1
AZD4573
All genes
-3kb TSS TTS 3kb
B
GATA-3
ITK
-log10 P value
LogFC
C
RNA processing
Ribosomes
DNA processing
Cell cycle
Transcription
Protein processing
Others
-logPvalue
D
Traveling ratio↑ (n=12998)
n=3045
Gene expression↓ (n=3901)
E
CDK9 target genes
ES=0.22
NES=1.3
FDR=0.05
PTCL
LN
CDK9 target genes
ES=0.35
NES=1.7
FDR=0.02
LCT
Non-LCT
F
GATA-3 target genes
G
IP
Input
IgG
GATA-3
IB
GATA-3
P300
GAPDH
J
sgGATA-3
GFP
GFP-GATA-3
75kD
25kD
50kD
GFP
GATA-3
GAPDH
H
GATA-3
CDK9
IKZF1
IKZF3
I
Pt2
Pt3
GATA-3
CDK9
CCNT1
ITK
TCF7
K
Ctrl
sgGATA-3
GFP
GFP-GATA-3
%input
satellite
ITK
GATA-3
RPB1
pS2
IgG
ns

**Supplemental Fig.S3. Changes in traveling ratio and binding profiles in CDK9 inhibited TCLs.** (A) Genome-wide occupancy heatmaps of RNA pol II binding peaks were shown in RNA pol II ChIP-seq profiles of H9 cells and PTCL, NOS patient specimens (Pt1) treated with DMSO and AZD4573 (50 nM for 4 hours). (B) Volcano plot of differential expressed genes from RNA-seq data of H9 cells treated with AZD4573 (50 nM for 4 hours). GATA-3 and representative GATA-3 target genes were labeled. (C) Enrichment analysis of CDK9 target genes was ranked by P value and shown with representative categories. (D) Venn diagram showing overlap of downregulated genes after AZD4573 treatment and genes have increased traveling ratio from ChIP-seq data in H9 cells treated with AZD4573 (50 nM for 4 hours). (E) Gene set enrichment analysis (GSEA) of CDK9 target genes in in-house CTCL and PTCL (GSE160119) gene expression profiling datasets. (F) Metagene occupancy heatmaps of RNA pol II binding peaks in GATA-3 target genes were shown in H9 cells and PTCL, NOS patient specimens (Pt1) treated with DMSO and AZD4573 (50 nM for 4 hours). (G) Endogenous GATA-3 and P300 are co-immunoprecipitated, as indicated, in H9 cells with DMSO and AZD4573 (50 nM for 4 hours). (H) Representative GATA-3 and CDK9 co-binding examples (IKZF1 and IKZF3 genomic loci) were shown in H9 cells. (I) Representative binding on GATA-3 target genes of GATA-3, CDK9 and CCNT1 ChIP-seq profiles were shown in two PTCL, NOS patient specimens. (J) GATA-3 expression was evaluated by IB in GATA3 knock out and restoration cells. Negative guide RNA and empty GFP were used as control, respectively. (K) GATA-3 (blue), RNA pol II (green) and pS2 (orange) and isotype (brown) binding on ITK promoter genomic loci were validated by ChIP-qPCR in Ctrl, sgGATA-3, GFP and GFP-GATA-3 H9 cells. Human satellite repeats was used a negative control. Data are represented as mean ± SEM. $^{*}p<0.05$, $^{**}p<0.01$, $^{****}p<0.0001$, ns, not significant (Welch's unpaired t-test; $n \geq 3$).

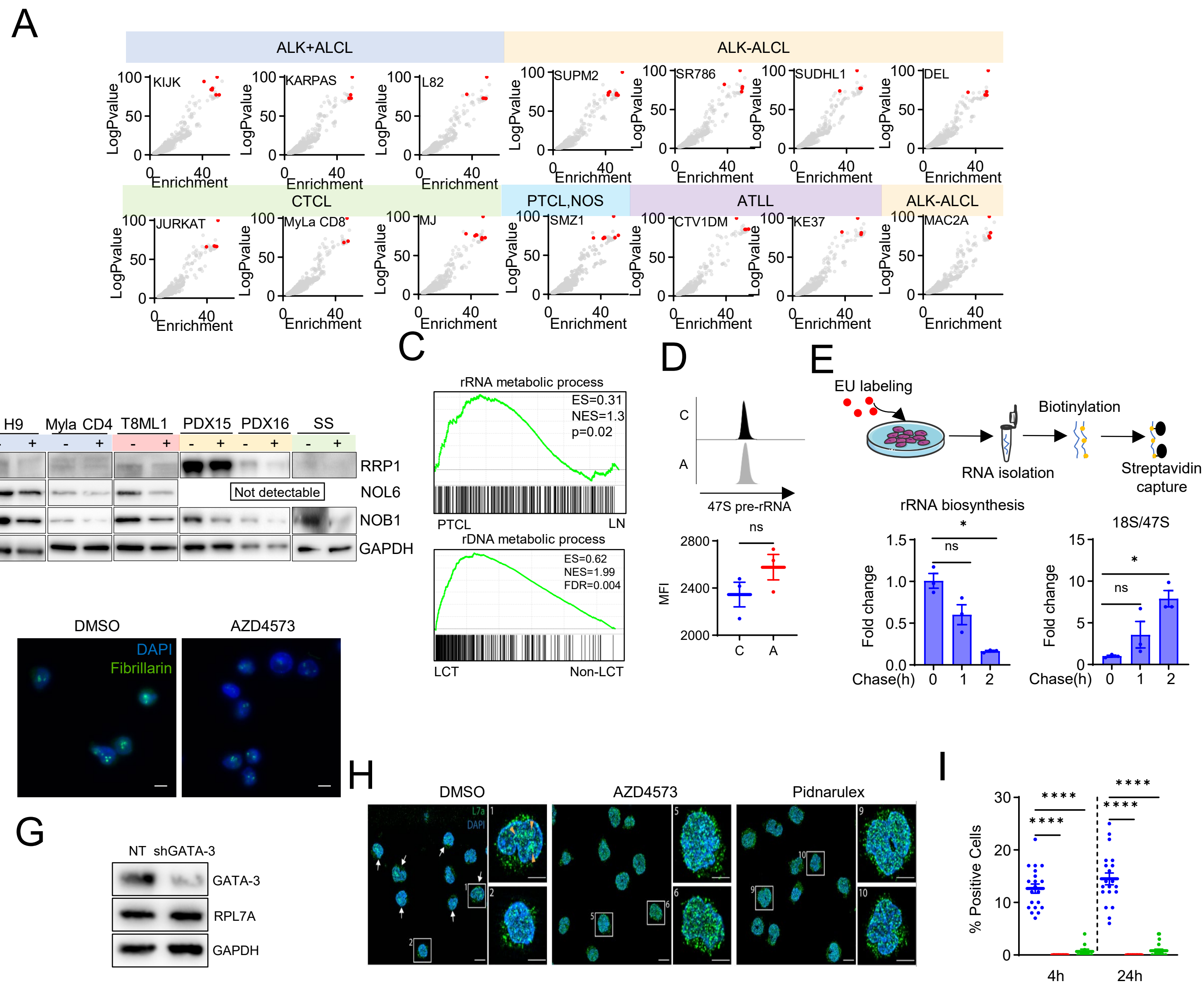
A
ALK+ALCL
ALK-ALCL
KIJK
KARPAS
L82
SUPM2
SR786
SUDHL1
DEL
CTCL
PTCL,NOS
ATLL
ALK-ALCL
JURKAT
MyLa CD8
MJ
SMZ1
CTV1DM
KE37
MAC2A
LogPvalue
Enrichment
B
H9
Myla CD4
T8ML1
PDX15
PDX16
SS
RRP1
NOL6
Not detectable
NOB1
GAPDH
C
rRNA metabolic process
ES=0.31
NES=1.3
p=0.02
PTCL
LN
rDNA metabolic process
ES=0.62
NES=1.99
FDR=0.004
LCT
Non-LCT
D
47S pre-rRNA
MFI
ns
E
EU labeling
Biotinylation
RNA isolation
Streptavidin capture
rRNA biosynthesis
18S/47S
Fold change
Chase(h)
ns
F
DMSO
AZD4573
DAPI
Fibrillarin
G
NT
shGATA-3
GATA-3
RPL7A
GAPDH
H
DMSO
AZD4573
Pidnarulex
I
% Positive Cells
4h
24h

**Supplemental Fig. S4. ribosome biogenesis is disrupted in CDK9 inhibition.** (A) Top 100 genes with most dependency score from DepMap CRISPR KO screens (24Q4) were selected for enrichment analysis in each TCL cell line. Pathways related to ribosome biogenesis were highlighted in red. (B) Expression of rDNA processing genes was determined by IB in AZD4573 treated cell lines (50 nM for 6 hours), a SS patient specimen (50 nM for 6 hours) and PDX cells in sacrifice day. (C) GSEA of genes related to rRNA metabolic process in in-house CTCL and PTCL (GSE160119) gene expression profiling datasets. (D) 47S pre-rRNA was measured by RNA FISH in DMSO (C) and AZD4573-treated (A) H9 cells, representative histogram was shown on the top and summary was shown on the bottom. (E) Cell-population-based RNA pulse-chase assays were used to assess pre-rRNA synthesis (47S pre-rRNA) and processing (ratio of 18S expression compared to 47S expression) in H9 cells. (F) Representative images of of Fibrillarin and DAPI staining shown in H9 cells treated with 50nM AZD4573 for 2 hours. Scale bar is 5 µm. (G) Expression of GATA-3 and RPL7A was determined by IB in negative control (NT) and GATA-3 knock-down (shGATA-3) H9 cell line after doxycycline induction. (H) Representative z-stacks of DeepSIM images of L7a and DAPI staining shown in H9 cells treated with 50 nM AZD4573 or 50 nM Pidnarulex for 4 hours. Cells with positive L7a phenotype are indicated with white arrows. Scale bar is 10 µm. Subpanel numbered 1 represents zoomed-in view of a cell with positive L7a phenotype. Nucleoli localization of L7a are indicated with orange arrows. Subpanels 2, 5, 6, 9 and 10 represent cells with negative phenotype (lack of nucleoli L7a localization). Scale bar is 5 µm. (I) L7a positive cells were summarized in 20 regions of interest (ROIs) by widefield microscopy in H9 cells treated with DMSO (blue), 50 nM AZD4573 (red), and 50 nM Pidnarulex (green) for 4 hours and 24 hours, respectively. Data are represented as mean ± SEM. *$p<0.05$, **** $p<0.0001$, ns, not significant (Welch's unpaired t-test; $n \geq 3$).
